# Developing Climate-Resilient Chickpea Involving Physiological and Molecular Approaches With a Focus on Temperature and Drought Stresses

**DOI:** 10.3389/fpls.2019.01759

**Published:** 2020-02-25

**Authors:** Anju Rani, Poonam Devi, Uday Chand Jha, Kamal Dev Sharma, Kadambot H. M. Siddique, Harsh Nayyar

**Affiliations:** ^1^ Department of Botany, Panjab University, Chandigarh, India; ^2^ Department of Crop Improvement Division, Indian Institute of Pulses Research, Kanpur, India; ^3^ Department of Agricultural Biotechnology, Himachal Pradesh Agricultural University, Palampur, India; ^4^ The UWA Institute of Agriculture, The University of Western Australia, Perth, WA, Australia

**Keywords:** chickpea, water limitation, high temperature, tolerance, genomics

## Abstract

Chickpea is one of the most economically important food legumes, and a significant source of proteins. It is cultivated in more than 50 countries across Asia, Africa, Europe, Australia, North America, and South America. Chickpea production is limited by various abiotic stresses (cold, heat, drought, salt, *etc*.). Being a winter-season crop in northern south Asia and some parts of the Australia, chickpea faces low-temperature stress (0–15°C) during the reproductive stage that causes substantial loss of flowers, and thus pods, to inhibit its yield potential by 30–40%. The winter-sown chickpea in the Mediterranean, however, faces cold stress at vegetative stage. In late-sown environments, chickpea faces high-temperature stress during reproductive and pod filling stages, causing considerable yield losses. Both the low and the high temperatures reduce pollen viability, pollen germination on the stigma, and pollen tube growth resulting in poor pod set. Chickpea also experiences drought stress at various growth stages; terminal drought, along with heat stress at flowering and seed filling can reduce yields by 40–45%. In southern Australia and northern regions of south Asia, lack of chilling tolerance in cultivars delays flowering and pod set, and the crop is usually exposed to terminal drought. The incidences of temperature extremes (cold and heat) as well as inconsistent rainfall patterns are expected to increase in near future owing to climate change thereby necessitating the development of stress-tolerant and climate-resilient chickpea cultivars having region specific traits, which perform well under drought, heat, and/or low-temperature stress. Different approaches, such as genetic variability, genomic selection, molecular markers involving quantitative trait loci (QTLs), whole genome sequencing, and transcriptomics analysis have been exploited to improve chickpea production in extreme environments. Biotechnological tools have broadened our understanding of genetic basis as well as plants' responses to abiotic stresses in chickpea, and have opened opportunities to develop stress tolerant chickpea.

## Introduction

Chickpea (*Cicer arietinum* L.) is the 2^nd^ most important legume crop after common bean (*Phaseolus vulgaris* L.) (Gaur et al., 2008; Varshney et al., 2013b) and an economically beneficial protein-rich food legume. India is the largest chickpea-producing country, with a 75% share of global production (FAO, 2016; Maurya and Kumar, 2018; Gaur et al., 2019). Chickpea is produced in 50 countries, of which Australia, Canada, Ethiopia, India, Iran, Mexico, Myanmar, Pakistan, Turkey, and the USA are the major producers (Gaur et al., 2012; Archak et al., 2016; Dixit et al., 2019). However, the productivity of chickpea is not sufficient to fulfill the protein requirement for the increasing human population (Henchion et al., 2017; Chaturvedi et al., 2018). Chickpea production faces many challenges due to various abiotic stresses such as drought, and low and high temperatures (Ryan, 1997; Millan et al., 2006; Gaur et al., 2008; Mantri et al., 2010; Jha et al., 2014; Garg et al., 2015). Most importantly, unpredictable climate change is the major constraint for chickpea production as it increases the frequency of drought and temperature extremes, *i.*e., high (> 30°C) and low (< 15°C) temperatures (Gaur et al., 2013; Kadiyala et al., 2016), which reduces grain yields considerably (Kadiyala et al., 2016). Thus, high- and stable-yielding varieties of chickpea during such stress conditions need to be developed (Chaturvedi and Nadarajan, 2010; Krishnamurthy et al., 2010; Devasirvatham et al., 2015; Devasirvatham and Tan, 2018).

Drought stress is a serious situation for agriculture in the context of climate change and the ever-increasing world population (Farooq et al., 2009; Tardieu et al., 2018). Extreme drought conditions reduce crop yields through negative impacts on plant growth, physiology, and reproduction (Yordanov et al., 2000; Barnabas et al., 2008). Across the globe, drought stress reduces chickpea yield by about 45–50% (Ahmad et al., 2005; Thudi et al., 2014). Numerous studies have been conducted on the drought effects on different chickpea traits, including early maturity, root traits, carbon isotope discrimination, shoot biomass (Kashiwagi et al., 2005; Krishnamurthy et al., 2010; Upadhyaya et al., 2012; Krishnamurthy et al., 2013b; Purushothaman et al., 2016), and morphological (Sabaghpour et al., 2006), physiological (Turner et al., 2007; Rahbarian et al., 2011), biochemical (Gunes et al., 2006; Mafakheri et al., 2010) and molecular traits (Mantri et al., 2007; Thudi et al., 2014; Garg et al., 2016). There have been various attempts to explain the advancements in “omics” technology for drought challenges. These advances should progress the development of stress-resilient, high yielding, and nutritionally superior varieties of chickpea.

Winter/autumn-sown chickpea crops in northern south Asia and south Australia face low temperature (LT) stress at reproductive (flowering/podding) stages whereas those in Mediterranean region, especially the central Anatolia, are exposed to LT at the seedling and early vegetative stages (Berger et al., 2005; Berger et al., 2011; Berger et al., 2012). Winter-sown crops in the West Asia and North Africa (WANA) or northern regions of south Asia flower when cold is over and temperatures rise. Podding temperatures are slightly higher than those for flowering (Berger et al., 2005), and flowers drop if temperatures remain lower than that required for podding. At flowering/podding time, the crop is also at the risk of damage by *Ascochyta* blight disease. A temperature of 14–6°C, usually 15°C, is considered a threshold for reproduction in chickpea (Srinivasan et al., 1998; Berger et al., 2004; Clarke et al., 2004; Berger et al., 2005; Bakht et al., 2006b; Berger, 2007), a recent study by Berger et al. (2012), however, measured mean flowering temperature to be 21°C which is well above the earlier estimates implying that most of the world chickpea is susceptible to cold stress. Winter sown chickpea is also prone to terminal drought, as delayed flowering extends the chickpea growing season to warm but low or no rainy periods. In contrast to this, spring sown crops in the Mediterranean, USA, and Canada are of short duration and do not face terminal drought but productivity is low due to short duration (Singh et al., 1997a). In USA, the rains may extend the crop growth season so long that crop fails to mature especially in the Montana region (McVay et al., 2013). Being a crop of indeterminate growth habit, drought conditions will hasten maturity in chickpea by stopping growth, while late season rains will cause plants to green back up (McVay et al., 2013).

Despite being a cool-season crop, chickpea also faces high-temperature (HT) stress during reproductive development in warmer regions and in late-sown environments. HT aborts floral buds, flowers, and pods, ultimately leading to reduced seed size and yield (Wang et al., 2006) especially those above 32°C (Kaushal et al., 2013; Devasirvatham et al., 2015). HT like LT leads to loss of pollen viability and pollen fertility that affect pod set (Wang et al., 2006; Kumar et al., 2013; Kaushal et al., 2016). HT induced disruption in sucrose synthesis and its availability to the anthers, and oxidative stress appears to contribute to loss of pollen fertility and stigmatic function (Kaushal et al., 2013; Kumar et al., 2013; Devasirvatham et al., 2015), resulting in poor pod set. Heat stress can have a highly destructive effect on grain growth and development in chickpea (Wang et al., 2006). The grain yield of chickpea is related to its phenology, which is influenced by temperature range (Jumrani and Bhatia, 2014). High temperatures (> 35°C) during the reproductive stage is a major constraint for chickpea productivity (Siddique et al., 1999; Wang et al., 2006; Basu et al., 2009), with temperatures >30°C reducing grain weight and number (Kobraee et al., 2010). Substantial reductions in chickpea yield have been observed for even a 1°C rise in temperature beyond the threshold (Kalra et al., 2008). Yield losses have increased to 100% in many chickpea genotypes, with increasing temperature (Canci and Toker, 2009). High temperature severely affects podding in chickpea; the magnitude of which may be due to impaired source and sink relations from green leaves to anther tissue that leads to the mortality of pollen grains (Awasthi et al., 2014). Heat stress after flowering and grain filling reduced chickpea yield, due to increased senescence and reduced grain set and grain weight per plant (Wang et al., 2006). Post-anthesis, both grain numbers and weight decreased at high temperatures, leading to lower grain yields (Summerfield et al., 1984; Wang et al., 2006; Devasirvatham et al., 2013). Heat stress, in future, would considerably reduce the grain yields in several crops, including chickpea, in many parts of the world, and thus deserves serious attention to develop heat-tolerant cultivars. Developing new cultivars with improved adaptation to high temperature is vital for increasing worldwide chickpea production.

Winter sown crops in all parts of world are prone to terminal drought, however, drought is not confined to terminal stages but it may occur at any plant growth stage. Spring-sown chickpea in WANA region and semi-arid tropics (SAT) faces drought at the vegetative as well as reproductive stages (Silim and Saxena, 1993) leading to 30 to 100% yield losses, depending on the genotype, and severity as well as timing of drought (Singh, 1993; Leport et al., 1999; Canci and Toker, 2009). Chickpea can tolerate drought stress based on “escape,” “tolerance,” and “avoidance” three important mechanisms (Levitt, 1972). The principle of drought escape constitutes completion of plant's life-cycle before the onset of drought stress by hastening the phenological events (Levitt, 1972; Berger et al., 2016). Drought avoidance mechanism features minimum water loss and maximizing water use (Levitt, 1972). Usually, under central and south Indian conditions where chickpea is grown under stored soil moisture and having high water holding capacity soil, chickpea withstands drought stress through employing drought escape and drought avoidance mechanisms (Berger et al., 2006; Berger et al., 2016). However, this drought avoidance strategy remains ineffective under Mediterranean climates in Western Australia featuring low water holding capacity soil (Berger et al., 2016). The sources of resistance to these stresses are available either in the cultigens (heat and drought stress) or wild relatives (cold stress), and can be exploited to develop stress-resilient chickpea cultivars. The methodologies may be as simple as hybridization to use of marker assisted breeding [for genes as well as quantitative trait loci (QTLs)] or development of transgenics. QTLs for drought and temperature tolerance and in several cases genes within QTL regions have already been identified (Varshney et al., 2013a; Varshney et al., 2016; Devasirvatham and Tan, 2018; Kaloki et al., 2019). Genic, genetic, physiological, and biochemical basis of stress tolerance, once explored sufficiently, are expected to form the guiding principles for development of stress management strategies in chickpea. The objectives of sustainability of chickpea productivity or enhancing it further under changing climates can not be achieved until chickpea cultivars tolerant to combined stress, such as drought and heat, and drought and cold are developed. Various defense mechanisms regulating chickpea's adaptation during temperature and drought stress, especially the combined stresses, also need to be investigated (Upadhyaya et al., 2012; Awasthi et al., 2015; Khan et al., 2019a; Khan et al., 2019b). Here, we update the research status on drought and temperature stress in chickpea, and suggest appropriate management strategies to develop stress-tolerant genotypes.

### Effects of Cold Stress

Chickpea (*C. arietinum* L.) has evolved in the Mediterranean region and developed sensitivity to low temperature, with adverse effects on growth and yield (Croser et al., 2003; Kaur et al., 2008a; Thakur et al., 2010; Kumar et al., 2013). About half of the productivity losses in chickpea are due to exposure to low temperature (Saxena, 1990). Chilling stress in chickpea mostly affects the northern parts of India and southern Australia, as temperatures drop below 15°C at flowering (Srinivasan et al., 1998; Clarke et al., 2004; Berger et al., 2006). The reproductive phase is critical for crop productivity (Thakur et al., 2010); chilling stress in chickpea causes flower abortion, pollen, and ovule infertility, disrupts fertilization, reduces pod set, retards seed filling, and reduces seed size and ultimately crop yield (Clarke and Siddique, 2004; Nayyar et al., 2005b; Nayyar et al., 2007; Thakur et al., 2010; Kumar et al., 2011). Low temperatures can limit chickpea growth and vigor at all phenological stages but are most damaging during the reproductive stage.

#### Germination and Vegetative Growth

Chickpea is a cool-season crop that is exposed to chilling (3–8°C) or even freezing temperatures during germination, which can affect seedling establishment and reduce seedling vigor (Chen et al., 1983; Srinivasan et al., 1998; Bakht et al., 2006b). Several interacting factors (genotype, temperature, duration and time of exposure, and seed moisture content prior to imbibition) mediate seed responses to low germination temperatures. Roberts et al. (1980) and Singh et al. (2009) demonstrated that low temperature (10°C) decreased the germination rate of chickpea seeds. The recommended threshold temperatures range for chickpea germination varies from 5 to 35°C and optimum germination temperature is 20°C (Singh and Dhaliwal, 1972; Ellis et al., 1986; Auld et al., 1988; Calcagno and Gallo, 1993). Chickpea, along with many other chilling-sensitive species, is prone to “imbibitional chilling injury” (Tully et al., 1981). In the field, chilled seeds are often vulnerable to infestation by soil organisms, which reduces seedling survival. Chen et al. (1983) observed that the greatest sensitivity to cold occurs in the first 30 min of imbibition in chickpea and low temperature (3 to 8°C) during imbibition reduced chickpea germination by 15%. The combination of imbibition at low temperature and fast water uptake reduced germination by 65% (Tully et al., 1981; Chen et al., 1983). In Australia, chilling damage during imbibition has been implicated in the poor establishment of some chickpea genotypes in cold and wet soils combined (Knights and Mailer, 1989). The rapidity of imbibition is a factor, controlled principally by the thickness of the testa (Tully et al., 1981; St. John et al., 1984). Kabuli types generally have thinner testa than desi types, resulting in more rapid imbibition of water and consequently greater levels of imbibitional damage.

Another factor affecting germination success at cold temperatures is the seed phenolic content (Auld et al., 1983; Wery, 1990), which presumably confers fungal properties (Wery et al., 1994). Thus, the poor germination of kabuli types is partly due to their thin white testa being more susceptible to soil pathogens. Cold stress adversely affects the mobilization of food reserves from cotyledons that decreases embryonic growth, germination, and growth of chickpea seedlings (Croser et al., 2003). Ellis et al. (1986) found genotypic differences in the rate of germination with temperature. Given the existing genetic variability, it should be possible to select genotypes that are resistant to temperature stress during germination. Some seed treatments, such as hydropriming for 12 h or osmopriming (PEG/0.5 MPa) for 24 h have increased germination of chickpea in low-temperature soil conditions (Elkoca et al., 2007), and may be linked to cross-tolerance. Chickpea plants growing under field conditions, especially in India and Australia, are exposed to gradually decreasing temperatures and photoperiods during the early vegetative stage (Croser et al., 2003). The minimum temperature that chickpea generally seems to survive is –8°C; however, some lines can tolerate as low as –12°C post-emergence (Wery, 1990; Croser et al., 2003). Thus, there is potential to select for cold tolerance at germination and during seedling growth from the existing chickpea germplasm.

#### Reproductive Growth and Yield

The flowering phase, the crucial phase in the plant life cycle that determines the yield of chickpea, is most sensitive to cold stress (Sharma and Nayyar, 2014). Temperatures below 15°C result in the abortion of chickpea flowers leading to decline in the number of pods per plant and seeds per pod (Srinivasan et al., 1999; Berger et al., 2004; Clarke and Siddique, 2004; Nayyar et al., 2005b; Berger et al., 2006; Kaur et al., 2011; Kumar et al., 2011). The causes of flower abortion in sensitive genotypes of chickpea are fairly well understood. It is well documented that male gametophyte of chickpea is highly sensitive to cold stress and in genotypes sensitive to cold, both microsporogenesis and subsequent pollen development are inhibited at temperatures below 10°C (Sharma and Nayyar, 2014; Kiran et al., 2019). Identification of flower and anther development stages in chickpea allowed studying the impact of cold at different flower development stages (Kiran et al., 2019). Flowers of different development stages react differently to cold stress (Kiran et al., 2019) e.g., low temperatures terminate microsporogenesis in flowers at pre-meiotic stage of anthers and microgametogenesis in those at tetrad stage. In anthers at young microspore stage, low temperatures inhibited anther dehiscence but did not inhibit development of microspores to mature pollen stage. The pollen, however, were sterile indicating that cold at this stage affected pollen viability, in addition to anther dehiscence (Oliver et al., 2007). Exposure at mature pollen stage delayed anther dehiscence and induced partial pollen sterility (Kiran et al., 2019). The quantum of low temperatures induced pollen sterility also depends upon the age of the flower with older flowers producing less amount of sterile pollen as compared to younger flowers, e.g., low temperature treatment at young microspore stage led to complete sterility of pollen whereas those at vacuolated microspore stage 23.59% pollen were viable, at vacuolated pollen stage 52.4% pollen were viable, at mature pollen stage 65.5% pollen were viable (Kiran et al., 2019). Apparently, male gametophytes of younger flowers are more prone to damage by cold stress as compared to the older ones. In contrast, cold-tolerant chickpea genotypes maintain functional anther and pollen development, leading to pod formation and seed set during chilling stress (Clarke and Siddique, 2004; Kumar et al., 2011). Cold stress also impairs pollen tube growth in the style and, consequently, fertilization failure (Clarke and Siddique, 2004; Nayyar et al., 2007).

Chilling stress also has an adverse effect on gynoecium to impair ovule function; Srinivasan et al. (1998) reported missing embryo sacs in some chickpea cultivars, which reduced the number of fertilized ovules in all cultivars during cold stress. Chilling stress reduces ovule viability, stigma receptivity, and pollen load on stigma (Kiran et al., 2019). While studying flower abortion due to cold stress in chickpea, it was observed that the older flowers, that have sufficient viable pollen were also aborted (Kiran et al., 2019). Very low ovule viability accompanied by very low stigma receptivity in older flowers pointed toward role of female gametophyte factors in lack of fertilization and flower abortion under low temperature stress in addition to male factors. The role of female gamete was also highlighted using pollen from cold treated flowers to pollinate plants growing at normal temperatures and *vice-versa* (Nayyar et al., 2005b). The low temperature (4°C) used by Kiran et al. (2019) was, however, considerably lower than the threshold of 15°C (Srinivasan et al., 1998; Clarke et al., 2004; Berger et al., 2004; Berger et al., 2005; Bakht et al., 2006b; Berger, 2007) or 21°C (Berger et al., 2012) reported for reproduction in chickpea. Further studies at temperature slightly below 15°C need to be conducted to understand behavior of flowers to threshold low temperature stress.

Ectopic persistence of tapetum in low temperature treated chickpea flowers indicates disruption of normal process of tapetum programmed cell death under low temperatures (Kiran et al., 2019). Such disruption might have imbalanced nutrition to developing microspores. It has been already documented that low temperatures during flowering cause nutritional deficiencies in the tapetum (Nayyar et al., 2005b; Sharma and Nayyar, 2014) and decrease in sugar levels in anthers and pollen grains, which may be a primary cause of flower abortion. Low temperatures disrupt the mobilization of carbohydrates from source to sink and lead to nutrient deficiencies in stylar tissues too (Nayyar et al., 2005b). Cold stress also induces the synthesis of abscisic acid (ABA) in chickpea flowers, indicating a correlation between flower abortion and high ABA concentration (Thakur et al., 2010). In chickpea exposed to low temperatures (12–15/4–6°C day/night), increased ABA concentrations caused flowers to abort (Nayyar et al., 2005a). ABA interferes with sucrose translocation to flowers (Kumar et al., 2010) probably by inhibiting sucrose transporter gene *invertase* as has been observed in crops like rice (Oliver et al., 2005; Sharma and Nayyar, 2016).

Chilling stress has a damaging effect on flower number, pod set, seed growth, and development in chickpea (Croser et al., 2003; Berger et al., 2004; Nayyar et al., 2005b; Thakur et al., 2010). Moreover, low temperature impairs seed filling processes, which reduces the size of chickpea seeds (Nayyar et al., 2005b; Nayyar et al., 2007; Kaur et al., 2008a). Grain yield is related to phenology of chickpea and a combination of low temperature induced factors i.e., poor plant growth, delay in flowering, flower abortion, delay in podding, pod abortion, and poor seed filling contribute to lower the yield of chickpea under cold (Berger et al., 2004). Poor pod set/filling as a result of cold stress is due to the disruption in photosynthesis and inhibition of translocation of initiating signals from leaves to the meristem or by changing plant architecture (Gogoi et al., 2018). The studies on estimation of yield losses in chickpea due to cold are scanty. Singh et al. (1993) grew cold tolerant and cold susceptible genotypes of chickpea both in spring (temperatures normal for crop) and autumn (temperatures stressful as low as −10°C) in Syria and compared yield among the genotypes and seasons. A highly cold susceptible chickpea line with cold rating of 7.8 (1 = no visible cold damage, 9 = all plants killed) yielded 161 kg/ha during winter (low temperature) season and 474 kg/ha during warmer spring season (Singh et al., 1993). In comparison to this, a line with cold rating of 5.2 yielded 632 kg/ha during winter season and 251 kg/ha during spring season (Singh et al., 1993) indicating that cold in susceptible genotypes caused huge yield losses. The spring season due to short duration, reduces productivity of chickpea as compared to longer winter seasons that allows more time for crop to grow and consequently higher yields. Nayyar et al. (2005c) reported 30% increase in seed yield per plant in glycine betaine (a compatible solute that accumulate in cold-tolerant plants in higher amounts under cold stress) treated plants over control in winter sown chickpea grown in low temperature prone northern regions of India (pot-based studies). Since, winter sown chickpea yields more as compared to spring sown one if genotype has adequate cold-tolerance, the emphasis worldwide is on development of cold tolerant cultivars of chickpea to increase productivity of the crop. Wild relatives of chickpea in primary gene pool (*Cicer reticulatum*, *Cicer echinospermum*) that are crossable with the cultigens are tolerant to cold can be ideal sources to introgression cold tolerance to chickpea for development of varieties for winter season (Berger et al., 2012).

#### Physiology

The physiological functions of plants are adversely influenced by low temperature (<20°C) (Thakur et al., 2010). Low temperatures (17.6/4.9°C; day/night for 26 days during reproductive phase) resulted in reduction in relative leaf water content, possibly due to a decline in root hydraulic conductivity, oxidative and membrane damage, and chlorophyll loss (Kumar et al., 2011). Chilling stress (13/10°C; day/night for 18 h) during germination considerably inhibited α-amylase activity, disrupted sugar metabolism, reduced leaf water status, and uptake of mineral elements (N, P, and K) that delayed seedling emergence and caused poor seedling growth in chickpea (Farooq et al., 2017). Temperature changes can impact root physiology, thus affecting ion absorption and may result in visible deficiency symptoms (Gregory, 1988). Low-temperature stress (5°C for 3 days) inhibited root growth and the capacity for water and mineral uptake to subsequently impact the nutritional influences on plant growth (Aroca et al., 2003; Heidarvand et al., 2011). Low temperatures (5/5°C for 4 days) also reduced the leaf water content because the stomata are unable to close (Lee et al., 1993; Farooq et al., 2009). Flower abortion and poor pod set in chickpea due to cold stress (12–15/4–6°C day/night during flowering stage) was attributed to decreasing levels of sucrose, glucose, and fructose in anthers and pollen in sensitive genotypes (Nayyar et al., 2005a). Endogenous proline and carbohydrates (glucose, rhamnose, and mannose) increased with cold stress (3°C for 7 days) in chickpea genotypes, and may play a role in osmoregulation and meeting the enhanced energy requirements (Saghfi and Eivazi, 2014); the cold-tolerant genotypes performed better in this regard.

#### Cellular and Physiological Mechanisms for Cold Survival

Low temperatures (0–10°C) result in rigidification of the plasma membrane that is sensed by plant cells (Yadav, 2010) to impair the integrity of phospholipids in the plasma membrane (Badea and Basu, 2009). In cold-tolerant chickpea genotypes, the content of unsaturated fatty acids increased during low-temperature exposure (10°C for 5 days followed by 4°C for 2 days) (Shahandashti et al., 2013), which possibly contributed toward maintenance of membrane integrity during cold stress. Mitochondria are the most vital cell organelles and play an important role in stress tolerance mechanisms by interacting with energy-dissipating elements such as alternative oxidase (AOX) (Borecky and Vercesi, 2005; Rurek et al., 2015). In optimum conditions, plant cells carry on the cytochrome-mediated pathway with the help of the mitochondrial electron transfer chain, which results in ATP synthesis by using the proton motive force (Dinakar et al., 2016). In unfavorable conditions, a new pathway is involved in which cytochrome reductase and cytochrome oxidases are replaced by AOX to protect respiration and metabolic processes. This suggests that mitochondria have the flexibility to alter their activities and enhance AOX activity during environmental stress (Shi et al., 2013; Vanlerberghe, 2013). There are different genes for AOXs, depending on plant species; for example, AOX in chickpea is encoded by the aox3 gene in mitochondria (Karami-Moalem et al., 2018), and might be involved in cold tolerance.

Reactive oxygen species (ROS) are produced in response to cold stress in chickpea (Kumar et al., 2011) and damage vital molecules in cells, including membranes. Generally, lipid peroxidation and hydrogen peroxide concentrations are measured as markers of temperature-induced oxidative stress (Awasthi et al., 2015). A positive correlation was observed between lipid peroxidation and malondialdehyde (MDA) concentration in *Cicer occidentalis* (Shahandashti et al., 2013). Plant cells have different mechanisms to combat oxidative damage by activating ant oxidative systems that include both non-enzymatic (e.g., tocopherols, ascorbate, proline) and enzymatic [e.g., superoxide dismutase (SOD), catalase (CAT), and ascorbate peroxidase (APX)] (Turk et al., 2014; Zouari et al., 2016). A few studies in chickpea have identified an increase in the double bond index due to enhanced lipoxygenase (LOX) activity, suggesting that increased LOX activity plays an important role in providing cold tolerance in chickpea (Padham et al., 2007; Wasternack, 2007; Pushpalatha et al., 2011). The up-regulation of various types of antioxidants has been correlated with cold tolerance in chickpea (Nayyar and Chander, 2004).

Some plant regulating molecules look promising for imparting stress tolerance (Bhandari et al., 2017), and have been investigated in chickpea for enhancing cold tolerance. Polyamines (PAs), with a polycationic nature at a physiological pH, bind strongly to the negative charges in cellular components such as nucleic acids, proteins, and phospholipids (Bouchereau et al., 1999) and interact with membrane phospholipids to stabilize membranes under stress conditions (Roberts et al., 1986). The depletion of PAs as a result of cold stress (5 to 25°C for 4 days) has been linked to the loss of flowers and pods (Nayyar and Chander, 2004). Exogenous application of PAs reduced H_2_O_2_ levels and MDA content and increased antioxidant levels in chickpea plants subjected to cold stress (Nayyar and Chander, 2004). Hence, it may be possible to improve cold tolerance in chickpea by increasing the content of PAs using genetic manipulation or exogenous application. Besides PAs, abscisic acid (ABA) is also involved in providing stress tolerance (Trivedi et al., 2016); cold-stressed (10–12/2–4°C day/night at bud stage) chickpea plants treated exogenously with 10 µm ABA had improved pollen viability, pollen germination, flower retention, and pod set (Kumar et al., 2008). At the cellular level, ABA-treated plants increased activities of SOD, catalase (CAT), ascorbate peroxidase (APX), ascorbic acid, glutathione, and proline. Trehalose, a disaccharide of glucose plays an important role as a compatible solute, stabilizes biological structures under abiotic stress (Jain and Roy, 2009), including dehydrated enzymes, proteins, and lipid membranes, and protects biological structures from damage during desiccation (Fernandez et al., 2010). It also acts as a membrane and molecule chaperone during water or cold stress (Crowe, 2007; Fernandez et al., 2010). Seed priming with trehalose reduced the oxidative damage to biological membranes and other vital organelles during cold stress (13/10°C for 18 h) in chickpea, and improved carbon assimilation, resulting in better seedling growth (Farooq et al., 2017). Increased accumulation of total and reducing sugars (especially trehalose) may protect against chilling stress by stabilizing cell membranes, ceasing protein denaturation and acting as a scavenger of free radicals (Benaroudj et al., 2001; Farooq et al., 2009).

Glycine betaine (GB), an amino acid, is a cryoprotective solute that protects the activities of enzymes and proteins and stabilizes membranes and photosynthetic apparatus under chilling (12–14/3–4°C day/night) and freezing temperatures at bud and pod filling stage (Rhodes and Hanson, 1993; McNeil et al., 1999; Nayyar et al., 2005c). Cold stress (12–14/3–4°C day/night at bud stage) decreased the endogenous GB concentration in chickpea leaves and flowers, resulting in the loss of pods (Nayyar et al., 2005c). Exogenously applied GB to chickpea plants at bud and pod filling stages during cold stress improved flower function, pollen germination, pollen tube growth, stigma receptivity, and ovule viability, leading to floral retention, pod set, and pod retention (Nayyar et al., 2005c). Moreover, treatment with GB at the pod filling stage improved seed yield/plant, number of seeds/100 pods. Cold tolerance induced by GB may be related to an increase in relative leaf water content (RLWC), chlorophyll and sucrose, and decrease in ABA and active oxygen species (malondialdehyde and hydrogen peroxide) (Nayyar et al., 2005b; Nayyar et al., 2005d; Nayyar et al., 2005e). Possible roles for GB in stress tolerance include stabilization of complex proteins and membranes *in vivo*, protection of transcriptional and translational machinery, and as a molecular chaperone for refolding enzymes (Rhodes and Hanson, 1993).

Cold stress is lethal to most plants; despite this, temperate plants survive the winter months through acclimation processes, which suggest that plant exposure to low but not freezing temperatures confers cold tolerance (Bohn et al., 2007). A comparative study on cold-acclimated (CA) and non-acclimated (NA) chickpea plants showed an increase in the ratio of unsaturated fatty acids and saturated fatty acids in CA plants (Kazemi-Shahandashti et al., 2014). Antioxidative enzymes, such as SOD, CAT, guaiacol peroxidase (GPX), and lipoxygenase (LOX), were highly active in CA plants and resulted in enhanced cold tolerance, compared to NA plants. The transcription levels of CaCAT and CaSOD genes were higher in CA plants than NA plants. Moreover, the transcription level of the Ca-Rubisco gene was higher in CA plants than NA plants. Thus, cold acclimation (23°C for 20 days, 10°C for 5 days, followed by −10°C for 15 min.) had a positive effect on chickpea plants during long-term cold stress (Kazemi-Shahandashti et al., 2014), and may be a critical means of increasing cold tolerance.

#### Genomics and Transcriptomics in Elucidating Molecular Responses of Chickpea Under Cold

The “omics” approaches such as genomics, transcriptomics, proteomics, and metabolomics have become integral part of scientific strategies to study regulation of plants' responses to abiotic and biotic stresses. Between the genomics and transcriptomics, genomics provide the knowledge of structure of the genome including genes, promoters, regulatory elements etc. whereas the transcriptome elucidate the functional component of genome at any stage of plant growth. Consequently, transcriptomics reveal changes, not only in the expression of genes in a plant under abiotic stresses but also the gene regulatory mechanisms that govern differential expression of genes. Transcriptomics also provide information on differences in gene regulation and expression between the tolerant and sensitive genotypes thereby depicting precisely the mechanisms that lead to tolerance or susceptibility. Such detailed information can also be used to understand coordination among different regulatory pathways and may be exploited in the agricultural crops to develop appropriate strategies to manage the abiotic stresses under field conditions. In chickpea, global transcriptome expression using complementary DNA-amplified fragment length polymorphism (cDNA-AFLP), differential display, or microarray techniques have been used to identify genes of potential importance for acclimatization/tolerance to cold and elucidate pathways regulating this process (Mantri et al., 2007; Dinari et al., 2013; Sharma and Nayyar, 2014). Using microarrays, 210 differentially expressed genes under cold were identified (Mantri et al., 2007). The cDNA-AFLP in association with 256 primer combinations revealed different transcript-derived fragments (TDFs) associated with cold in chickpea leaves (Dinari et al., 2013). Some of the TDFs showed a differential expression pattern and belonged to putative functions associated with transport, signal transduction pathways, metabolism, and transcription factors. Various genes are activated in chickpea during low-temperature stress, which encode for transcription factors and components involved in detoxification processes and cell signaling. For example, the gene encoding phosphatidylinositol-4-kinase, a key enzyme in an influx of Ca^2+^ into the cytoplasm, expressed in Jk649809 and Jk649838 chickpea genotypes, (Scebba et al., 1998). The mitogen-activated protein kinase was also up-regulated in Jk649803 during cold acclimatization and might be a signal molecule for cold tolerance. It was concluded that cold tolerance in chickpea is regulated by a relatively small number of genes (Dinari et al., 2013).

Transcriptome analysis of meiotic anthers of chickpea revealed that cold-tolerance-associated genes belonged to four main categories—carbohydrate/triacylglycerol metabolism, pollen development, signal transduction, and transport (Sharma and Nayyar, 2014). All of the genes of these four categories were upregulated in cold-tolerant anthers, with the exception of one pollen development gene that was down-regulated. Genes involved in microspore/pollen growth (tetrad separation, pollen expansion, increased vascular transport, fatty acid transport, pollen maturation, pollen exine formation, pollen tube growth, fertility, and pollen development) were switched-on in cold-tolerant genotype under cold stress (Sharma and Nayyar, 2014). Upregulation of genes associated with carbohydrate and triacylglycerol metabolism suggests that cold-tolerant chickpea plants produce viable pollen during chilling stress by maintaining pollen development and carbohydrate/triacylglycerol metabolic pathways (Sharma and Nayyar, 2014). Another study reported increased expression of 109 and 210 genes when chickpea was exposed to drought and cold stress, respectively (Mantri et al., 2007). Of these, 15 and 30 genes were differentially expressed between tolerant and sensitive genotypes, respectively, which coded for various regulatory and functional proteins. Significant differences were observed in stress responses within and between tolerant and susceptible genotypes indicating multi-gene control and a complex abiotic stress response mechanism in chickpea. This study demonstrated that the leaves of cold-tolerant chickpea over expressed serine/threonine protein kinase while the flowers of cold-sensitive chickpea up-regulated SOD, a copper chaperone precursor involved in oxidative stress. Auxin repressed protein (DY475078) and auxin-responsive protein IAA9 (DY396315) transcripts, which are involved in cell rescue, were induced in the flowers and leaves of both the sensitive genotypes. Two phosphate-induced proteins (DY475076 and DY475172) were induced in flowers/pods of tolerant-1 (Sonali) chickpea genotype (Mantri et al., 2007). It is worth mentioning here that phosphorus is responsible for flower formation and seed production. Sucrose synthase (DY475105) was also induced in leaves of Sonali, which lead to the accumulation of sucrose that functions as an osmolyte and may provide cold tolerance.

To compare similarities and differences between cold-stressed anthers and gynoecium, a small subset of 25 genes that were up-regulated in anthers under cold, was used to study gene expression in gynoecium (Sharma and Nayyar, 2014). While all the genes were expressed in both the organs, nine had contrasting expression patterns in both the organs, i.e., an increase in one organ and decrease in the other (Sharma and Nayyar, 2014). The genes expressed under cold were also compared with those expressed under drought and salinity (Mantri et al., 2007). Some of the genes were common between the stresses while others were unique (Mantri et al., 2007; Mantri et al., 2010), which suggests that some segments of abiotic stress responsive machinery are shared by different abiotic stresses.

Whole genome sequencing (WGS) has also provided insights into cold-tolerance mechanisms in chickpea. The technique has been exploited to generate genomic resources for better understanding of cold-tolerance and cold-susceptibility in chickpea, such as identification of a flowering repressor gene *MtVRN2* in the conﬁdence interval of a QTL (Mugabe et al., 2019), using the reference genome of CDC Frontier chickpea. GWS has also been used to identify mitogen-activated protein kinases (MAPKs) in chickpea and the impact of cold on their expression. Of the 19 MAPK genes detected in chickpea, 15 were induced by low temperature (4°C, chilling stress) compared to control plants (Singh et al., 2018). Similarly, 36 genes encoding the K^+^ transport system in the chickpea genome were identified, along with their promoters with putative cold signals (Azeem et al., 2018). These studies provided new vital information about the genes, which might be associated with cold tolerance to chickpea and indicated that cold-tolerance mechanisms might have organ specific distinctions e.g., leaf, anther and gynoecium. To confirm association of these candidate genes in cold tolerance or cold susceptibility, further studies need to be conducted using appropriate models.

There is also a study indicating that changes in methylation patterns may be associated with cold tolerance in chickpea. Prolonged cold stress in a cold-tolerant genotype increased demethylation, relative to a cold-susceptible genotype, suggesting a higher potential for activation of cold-stress-responsive genes (Rakei et al., 2016). Thus, WGS and its further exploitation has generated genomic resources and enhanced our understanding of mechanisms governing cold tolerance/susceptibility in chickpea. These resources are ideal starting points for subsequent studies aimed at the regulation of cold tolerance in chickpea. The recent description of flower and anther development stages in chickpea (Kiran et al., 2019) is also expected to aid in the identification of molecular mechanisms for cold tolerance during different stages anther development.

Physiological studies (see previous sections for details) point to prominent role of carbohydrate metabolism, antioxidants, and free amino acids in cold-tolerance, however, gene regulatory networks for carbohydrates, antioxidants, and free amino acids under cold-tolerance have not been studied in detail. To understand intricacies and reveal complete picture of cold-susceptibility or tolerance in chickpea, merger of physiological and gene regulation knowledge under cold stress is essential. There is also a need to generate information on gene regulation/expression for antioxidants, carbohydrates, and free amino acids where physiological studies have already been conducted. Since, mechanisms of cold-tolerance by leaves may be different from flowers, which are complex organs involving microsporogenesis, microgametogenesis, megasporogenesis, pollination, fertilization, and seed development (Kiran et al., 2019), studies also need to be launched to understand mechanisms of pollen viability/ovule viability under cold stress by the cold-tolerant genotypes.

#### Genetic Variability and Breeding for Cold Tolerance

Winter-sown chickpeas face cold stress during reproductive growth resulting in flower drop, pod drop, and poor seed set (India and Australia) and restricted vegetative growth in young plants (Mediterranean region) (Singh et al., 1989; Saxena, 1990; Chaturvedi et al., 2009; Sharma and Nayyar, 2014; Sharma and Nayyar, 2016). The cold environment differs in these chickpea cultivation areas; temperatures remain subzero (freezing) for some time during early crop growth in the Mediterranean region but usually above zero in Indian and Australian regions. Consequently, the goals of cold-tolerance breeding will vary between regions, i.e., genotypes should be selected for freezing tolerance (below 0°C) during early growth in the Mediterranean region and chilling tolerance (up to 0°C) during reproductive growth in Indian subcontinent (Chaturvedi et al., 2009). Screening scales based on plant death at subzero temperatures are well described for cold-tolerant chickpea germplasm (Singh et al., 1989 [1–9 scale]; Saccardo and Calcagno, 1990 [0–5 scale]). However, no screening scales have been devised to identify chilling tolerance during reproductive growth, and appears to be due to the complexity of processes at reproductive phase (flowering, podding, seed set, seed development, etc.) and mechanisms by which cold impedes flower, anther, and pod development (Sharma and Nayyar, 2014; Kiran et al., 2019). Moreover, temperature sensitivity varies for flower, pod, and seed growth. For example, the critical temperature for seed growth is higher than that required for pod set (Srinivasan et al., 1998). Evidence is emerging that pod set is related to cumulative temperature rather than minimum temperature, as plants growing at 0°C night temperature and 20°C day temperature bore pods (Srinivasan et al., 1998). These observations need to be confirmed, as an earlier study reported that pod set only occurred at minimum night temperatures above 8°C (Saxena, 1990).

Several studies have been undertaken on freezing tolerance in the cultigens or *Cicer* species. Within *C. arietinum*, germplasm including M 450, ILC 8262, ICCV 88501, ICCV 88502, ICCV 88503, ICCV 88506, FLIP 84-70C, FLIP 84-71C, and FLIP84-79 C are tolerant to cold (Singh et al., 1990; Singh and Saxena, 1993) along with FLIP 81-293C, FLIP 82-127C, FLIP82-128C (Wery, 1990), ILC 8262 (a germplasm line), ILC 8617 (a mutant) and FLIP 87-82C (a breeding line) (Singh et al., 1995), ICCV 88501 and ICCV 88503 (Srinivasan et al., 1998), FLIP95-255C, FLIP93-260C and Sel95TH1716 (Kanouni et al., 2009), and Sel96TH11404, Sel96TH11439, Sel96TH11488, Sel98TH11518, x03TH21, and FLIP93-261C (Saeed et al., 2010). Freezing tolerance in chickpea is dominant over susceptibility and controlled by at least five sets of genes (Malhotra and Singh, 1990). Further genetic analysis revealed the presence of genic interactions (additive × additive and dominance × dominance) with duplicate epistasis and additive gene effects (Malhotra and Singh, 1991). The two types of chickpeas, desi, and kabuli, do not differ in their reaction to cold (Berger et al., 2012).

There is growing evidence that wild relatives of chickpea possess a higher degree of cold tolerance than the cultigens (Singh et al., 1995; Berger et al., 2012). Wild *Cicer* species of the primary gene pool are readily crossable to the cultigens and can be the potential donors of cold tolerance. Wild species were evaluated extensively for cold tolerance both at freezing (young plants) and to a limited extent in chilling environments (at the reproductive stage). Among the wild relatives, *Cicer bijugum, C. echinospermum*, and *Cicer judaicum* were more cold-tolerant than *C. arietinum* during early growth (Singh et al., 1990; Malhotra, 1998) of the reproductive stage (Berger et al., 2012). Among 59 lines from seven annual wild *Cicer* species, 26 lines of *C. reticulatum*, 10 of *C. bijugum*, 4 of *C. echinospermum*, 2 of *Cicer pinnatifidum*, and 1 of *C. judaicum* tolerated freezing (subzero conditions) during early vegetative growth (Singh et al., 1995). Among the cold-tolerant wild species, five lines of *C. bijugum* and four of *C. reticulatum* (highly tolerant) were superior to the cultigens for cold tolerance. In another study, Toker (2005) evaluated 43 accessions of eight annual wild *Cicer* species (*C. bijugum, Cicer chorassanicum, Cicer cuneatum, C. echinospermum, C. judaicum, C. pinnatifidum, C. reticulatum,* and *Cicer yamashitae*) for cold tolerance in young plants at subzero temperatures (freezing tolerance). *C. bijugum* was the best source of cold tolerance, with all six accessions under study being cold-tolerant (AWC 6: free from any damage, AWC 2 and AWC 4: highly tolerant, AWC 1, AWC 3, and AWC 5: tolerant) (Toker, 2005). Eleven of 15 accessions of *C. reticulatum*, 4 of eight *C*. *echinospermum*, and 1 of five *C. pinnatifidum* (score 3) were cold-tolerant.

Chilling-tolerant chickpea germplasm—CTS 60543 (ICCV88516), CTS11308 (ICCV88510)—has been identified (Clarke and Siddique, 2004). Pollen selection [transfer of plants to cold stress (12/7°C) for 3 days immediately after pollination followed by F_1_ seed collection] was used to develop chilling-tolerant chickpea varieties including Rupali (WACPE 2095) and Sonali (WACPE 2075) (Clarke et al., 2004). Similar to freezing stress, accessions of *C. arietinum* had less chilling tolerance than wild accessions (Berger et al., 2012). Even Rupali and WACPE 2078 developed by Clarke et al.(2004), when grown at ∼10°C post-anthesis, had large ﬂower–pod intervals (>65 days) indicating a low degree of cold tolerance (Berger et al., 2006). Among the wild species, an accession of *C. echinospermum* had robust chilling tolerance, whereas JM2106 of *C. reticulatum* was also chilling tolerant (Clarke and Siddique, 2004; Berger et al., 2012). The *C. echinospermum* accession not only expressed the early podding character at low temperature but also yielded five times more than the most productive chickpea cultivar. With duplications in gene bank accessions of wild species of *Cicer* (Croser et al., 2003), the actual number of cold-tolerant sources may be lower than that reported in the literature. Nonetheless, wild *Cicer* species are important sources for improving cold tolerance in chickpea.

One of the major consequences of low temperature has been hypothesized to be low sink utilization in northern regions of India, where low temperature causes flower abortion or failure of set pods (Saxena et al., 1988). To improve harvest index due to pod set failure in this region, chilling-tolerant lines were crossed with agronomic ally desirable lines (Saxena et al., 1988). Early flowering and podding in cross bred lines improved harvest index (50–54%) more than late flowering lines (39–42%). Cold-tolerant wild species of *Cicer,* namely *C. reticulatum* and *C. echinospermum,* have also been exploited to develop high-yielding chickpea (Singh and Ocampo, 1997). Cold-tolerant and *Fusarium* wilt resistant accession of *C. reticulatum* (ILWC 124) and *C. echinospermum* (ILWC 179) were crossed with cultigens (ILC 482); one of the progenies out-yielded ILC 482 by 39%. In another study, lines derived from a cross of cultivated chickpea and *C. reticulatum* out-yielded the check cultivars (Singh et al., 2005). Both studies showed that wild *Cicer* is not only a source of tolerance for abiotic stresses and diseases but can contribute to yield enhancement in chickpea. Both chilling tolerance during reproductive growth and yield enhancement in pedigree lines indicate that wild species of the primary gene pool have the potential to increase chickpea productivity in Australia and the Indian subcontinent (the region with the maximum area under chickpea) where cold stress coincides with the reproductive phase of the crop and productivity is low.

#### Genomics Advancements for Developing Cold Stress Tolerance in Chickpea

Generation of adequate genomic resources such as simple sequence repeat markers (SSRs) and single nucleotide polymorphism (SNPs) is essential for gene/QTL mapping and for identifying genes in QTL intervals. Currently available bioinformatics tools allow identification of molecular and biological functions of genes in QTL intervals based on existing scientific information, thereby allowing the selection of candidate genes governing the trait. The gene linked markers or QTLs can also be used to identify introgression of gene(s) into elite cultivars using a technique called foreground selection and recovery of recurrent parent genome using the background selection. Our understanding of cold tolerance in chickpea has increased considerably in the last decade, primarily due to advances in sequencing technologies that enabled large-scale decoding of genomic sequences at lower cost leading to gene identification, gene regulation, or large-scale development of DNA-based markers such as simple sequence repeats (SSRs) and single nucleotide polymorphism (SNPs). Development of reference genome sequences in chickpea (Jain et al., 2013; Varshney et al., 2013b; Parween et al., 2015) provided the much needed push in advancement of genomic resources in chickpea including development of SSR or SNP markers, identification of candidate genes within QTL intervals. Marker developments have allowed identification of QTLs governing tolerance to abiotic stresses. Association mapping of a panel of 44 genotypes was used to identify QTLs associated with freezing tolerance; however, no QTL associated with cold tolerance could be identified (Saeed and Darvishzadeh, 2017). The lack of adequate marker density appears to explain the non-detection of QTLs linked to cold tolerance as only 64 AFLP markers were used. Recently, a mapping population of 129 recombinant inbred lines (RILs), derived from an interspecific cross between ICC 4958 (cold-sensitive, desi type, *C. arietinum*) and PI 489777 (cold-tolerant wild relative, *C. reticulatum* Ladiz), followed by genotyping-by-sequencing was used to identify QTLs linked to cold tolerance (Mugabe et al., 2019). A total of 747 SNP markers, spanning 393.7 cM, were used in this study. The SNPs were more abundant than traditional markers and had considerably higher marker density, with an average of 1.8 SNPs cM^−1^. Freezing tolerance in PI48977 was governed by three QTLs situated on linkage groups (LGs) 1B, 3, and 8 (Mugabe et al., 2019); CT Ca-3.1 (on LG3) and CT Ca-8.1 (on LG8) were more important and accounted for 34 and 48% of the phenotypic variance for cold, respectively. One of the parents used in the study, *C. reticulatum*, requires vernalization, i.e., acceleration of flowering following brief spells of cold exposure (van Oss et al., 2015) and QTLs for vernalization response were also identified using a RIL population where one of the parents was PI 489777 (Samineni et al., 2016). It is worth mentioning here that cultigen, *C. arietinum*, does not respond to vernalization (Berger et al., 2005. Using 1,291 loci [SSRs, diversity array technology (DArT), cleaved amplified polymorphic sequences (CAPs), legacy markers, etc.] for QTL identification, a major vernalization response QTL was identified (Samineni et al., 2016). The QTL spanned 22 cM on LG3 and explained 47.9 to 54.9% of the phenotypic variation. Both studies, Samineni et al.(2016) and Mugabe et al.(2019) used the same cold-tolerant and vernalization responsive parent (PI 489777), and identified the same QTL (CT Ca-3.1) linked to the cold tolerance and vernalization response. This finding necessitates further research to determine the relationship between cold tolerance and vernalization response machinery in *Cicer* species. Using CDC Frontier chickpea as a reference genome, a homolog of the *Medicago truncatula* vernalization gene named *VERNALISATION2‐LIKE*VEFS box gene (*MtVRN2*) was mapped in CTCa-3.1 conﬁdence interval (Mugabe et al., 2019). *MtVRN2* is a repressor of the ﬂowering locus T gene homolog from *M. truncatula* and is a repressor of transition to flowering (Jaudal et al., 2016). This example demonstrates that genome sequences can be exploited effectively to narrow possible candidate genes in QTL regions and vernalization response in *Cicer* might be inversely related to flowering. None the less, QTLs governing cold tolerance in chickpea or candidate cold tolerance genes within these intervals are poorly explored so far as no information is available for QTLs in other cold-tolerant genotypes of *C. reticulatum*. Moreover, QTLs for cold-tolerance within cold-tolerant genotypes of *C. arietinum* and another annual wild relative *Cicer echnospermum* that possesses tolerance to cold are yet to be identified. In addition, no efforts have so far been made to transfer cold-tolerance QTLs from *C. reticulatum* to *C. arietinum*.

### Impacts of Heat Stress

Excessive heat stress affects all aspects of chickpea growth, phenology, and development (Devasirvatham et al., 2012; Devasirvatham et al., 2013; Kaushal et al., 2013), including biomass, flowering duration, pod number, days to maturity, seed weight, and grain yield (Upadhyaya et al., 2011; Kaushal et al., 2013) and a wide range of plant development and physiological processes. The impact of heat stress at different stages of plant growth and development in chickpea are described below.

#### Germination and Vegetative Growth

High temperatures affect seed germination in chickpea; genotypic variation was observed for high-temperature tolerance at seed germination, with no germination above 45°C (Singh and Dhaliwal, 1972; Ibrahim, 2011), reduced seedling growth (Kaushal et al., 2013), and even seedling death (Kaushal et al., 2011). Controlled environment studies showed significant biomass increases in both tolerant and sensitive genotypes at 35/25°C whereas exposure to 40/30°C decreased biomass at maturity in all genotypes, more so in the sensitive genotypes (Kumar et al., 2013).

#### Reproductive Growth

Heat stress limits chickpea growth and vigor at all phenological stages, but the reproductive phase is considered more sensitive to temperature extremes than the vegetative stage (Sita et al., 2017). Heat stress during reproduction generally 1) reduces flower number, 2) increases flower abortion, 3) alters anther locule number decrease, 4) causes pollen sterility with poor pollen germination, 5) reduces fertilization and stigma receptivity, 6) causes ovary abnormalities, 7) reduces the remobilization of photosynthates to seeds, and 8) reduces seed number, seed weight, and seed yield (Devasirvatham et al., 2012; Devasirvatham et al., 2013; Kaushal et al., 2013). Exposure of chickpea to heat stress (35/20°C) pre-anthesis reduced anther development, pollen production, and fertility by inducing physiological abnormalities (Devasirvatham et al., 2012). High temperature can induce anther and pollen structural aberrations, such as alterations in anther locule number, anther epidermis wall thickening, and pollen sterility, which are key factors reducing chickpea yield under high temperature (Devasirvatham et al., 2013). In chickpea, pollen is more sensitive to heat stress than the female gametophyte (Devasirvatham et al., 2012). The effect of high-temperature stress post-anthesis has been associated with poor pollen germination, pollen tube growth and fertilization, and the loss of stigma receptivity (Kaushal et al., 2013; Kumar et al., 2013), which reduces seed number, seed weight, and seed yield (Summerfield et al., 1984; Wang et al., 2006). Temperatures above 45°C are detrimental to pollen fertility and stigma function in chickpea (Devasirvatham et al., 2015).

Heat tress enhanced oxidative stress and lowered leaf photosynthesis, which reduced the soluble carbohydrate and ATP contents in the pistil (Kumar et al., 2013) and prevented nutrient transport from the style to pollen tube thus inhibiting pollen tube growth and ovary development (Kumar et al., 2013). Screening chickpea genotypes for heat sensitivity revealed substantial genetic variation in a high-temperature environment (Krishnamurthy et al., 2011; Devasirvatham et al., 2015). Heat-tolerant chickpea genotypes produced pods at temperatures above 35/20°C, while sensitive genotypes aborted most of their flowers (Kaushal et al., 2013). Devasirvatham et al. (2013) reported greater pod set in heat-tolerant genotypes (ICC 1205 and ICC 15614) than heat-sensitive genotypes (ICC 4567 and ICC 10685).

#### Influence of Heat Stress on Physiology

Some vital physiological traits, including chlorophyll concentration, photosynthetic rate, and membrane stability of leaf tissue, can be used as indicators of heat sensitivity (Hasanuzzaman et al., 2013). Chickpea is relatively more sensitive in terms of membrane stability and photosystem II function at high temperatures 50°C for 48 h than other legumes (Srinivasan et al., 1996). Heat stress (35/16°C for 10 days) induces leaf senescence in chickpea (Wang et al., 2006) by disrupting the chloroplasts and damaging chlorophyll. Heat stress (>32/20°C during reproductive stage) reduced the chlorophyll content in chickpea leaves, which caused chlorosis (Kaushal et al., 2013); this loss may have occurred due to photo-oxidative stress or inhibition of chlorophyll synthesis (Guo et al., 2006). Heat stress (>32/20°C during reproductive stage) caused more leaf damage in a heat-sensitive than heat-tolerant chickpea genotype, due to a greater reduction in leaf water status (as RLWC) and possible decline in stomatal conductance, and restriction in hydraulic conductivity of root (Kaushal et al., 2013). Transpiration efficiency in chickpea decreased with increasing temperature (Singh et al., 1982). The quantum yield or photosystem II (PSІІ) activity in chickpea was not affected at 35°C, but a noticeable reduction occurred at 46°C (during pod filling) that caused irreversible damage to photosynthetic systems (Basu et al., 2009). Similarly, Srinivasan et al. (1996) reported severe damage to PSІІ at 50°C for 48 h in chickpea. Temperatures above 35°C during reproductive stage suppressed photosynthesis and electron flow and disrupted metabolic pathways to reduce grain size (Kaushal et al., 2013; Awasthi et al., 2014; Redden et al., 2014).

Heat stress alters the fluidity of plasmalemma, mitochondria, and chloroplast membranes, which can disintegrate the lipid bilayer to change the protein conformation and cause protein unfolding (Pastor et al., 2007). Heat stress also results in the production of ROS that damage photosynthetic apparatus and other components, thus hampering metabolic activity (Allakhverdiev et al., 2008; Das and Roychoudhury, 2014). Respiration is more temperature-sensitive than photosynthesis (Hatfield et al., 2011). At 45/35°C (day/night), the cellular oxidizing ability of chickpea plants reduced appreciably at vegetative stage (Kumar et al., 2013), suggesting impaired respiration and energy generation, possibly due to the inactivation of enzymes (Salvucci and Crafts-Brandner, 2004).

At high temperature (> 32/20°C), sucrose synthesis decreased due to the inhibition (40–43%) of sucrose synthesizing enzymes (sucrose synthase and sucrose phosphate synthase) to impair sucrose metabolism in leaves of chickpea during reproductive phase (Kaushal et al., 2013). As a result, the sucrose flow to flowers in heat-sensitive genotypes was considerably decreased to affect the developmental and functional aspects of pollen grains resulting in poor fertilization and pod set (Kaushal et al., 2013). High temperatures (32/20°C day/night) from anthesis to maturity reduced starch deposition in chickpea grains because of reduced activity of ADP-glucose pyrophosphorylase and starch synthase (Vu et al., 2001; Awasthi et al., 2014) resulting in reduction in grain weight.

#### Cellular Mechanisms for Survival Under Heat

Under heat stress (>35/23°C day/night) at the time of flowering, chickpea experiences adverse effects on growth and various metabolic processes that lead to alterations in the redox state of the cell (Kaushal et al., 2011; Awasthi et al., 2015). At high temperature (37 and 42°C for 10 h), ROS generation causes oxidative damage to vital cellular components, such as membrane lipids, proteins, nucleic acids, pigments, and enzymes (Rivero et al., 2001; Suzuki and Mittler, 2006; Yin et al., 2008). The ROS-induced oxidative damage consists of both free radicals, including hydroxyl radicals (OH˙), superoxide (O_2_^−^), alkoxyl radicals, and non-radicals like hydrogen peroxide (H_2_O_2_) and singlet oxygen (^1^O_2_) (Suzuki and Mittler, 2006). At 40/30 and 45/35°C during growth and germination stage, increased lipid peroxidation and hydrogen peroxide levels in the leaves of heat-sensitive chickpea genotypes caused more leaf damage, than in tolerant genotypes (Kaushal et al., 2011; Kumar et al., 2012b; Kumar et al., 2013). Heat tolerance mechanisms in chickpea are potentially characterized by higher levels of antioxidants and osmolytes (Kaushal et al., 2011), which maintain membrane integrity, protect macromolecules, and sustain metabolism, leading to heat acclimatization. Under stressful conditions, plants tend to combat ROS production by inducing an antioxidant system consisting of enzymatic and non-enzymatic components (Gill et al., 2012); for example in chickpea, the activities of SOD, catalase (CAT), and ascorbate peroxidase (APX) increased at 40/35°C during growth and germination stage but decreased at 45/40°C (Kaushal et al., 2011). Similar, the activity was observed in non-enzymatic antioxidants ascorbate (ASC) and glutathione (GSH). Inhibition of these enzymes and non-enzymatic antioxidants was much more in the heat-sensitive genotypes: the antioxidants increased at 40/35°C but declined at 45/40°C observed (Kaushal et al., 2011) in heat-sensitive genotypes. Exogenous application of proline (Pro), an osmolyte, significantly increased SOD, CAT, ASH, and GSH activity at 45/40°C in chickpea, relative to the plants grown without proline (Kaushal et al., 2011).

Salicylic acid (SA) plays a key role in providing tolerance against temperature stress in chickpea. Heat-stress-induced membrane damage in chickpea plants declined significantly with the application of SA, relative to the untreated control and heat-acclimatized plants (Chakraborty and Tongden, 2005). The SA treatment also altered the contents of proteins and proline, significantly with induction of various stress enzymes such as peroxidase (POX), ascorbate peroxidase (APOX), and catalase (CAT) activities (Chakraborty and Tongden, 2005). Abscisic acid also appears to be involved in thermotolerance of chickpea; exogenous ABA application (2.5 μM) at 4 day seedling significantly alleviated the effects of heat stress (45/40°C for 10 days) in chickpea (Kumar et al., 2013) by improving plant growth and reducing oxidative damage. Another study showed that exogenous nitrogen application during pre-flowering and suitable irrigation helped to mitigate the effects of heat stress (>35°C) in chickpea (Upadhyaya et al., 2011). Heat stress (38°C for 10 days) induced the accumulation of raffinose family oligosaccharides (RFOs), such as galactinol and raffinose; galactinol synthase (GolS) is a key regulatory enzyme of RFO biosynthesis. In a recent study, galactinol and raffinose content increased significantly in response to heat stress in chickpea (Salvi et al., 2017).

During heat stress, heat shock genes encode different heat shock proteins (HSPs), which accumulate and protect cells by acting as molecular chaperones (Huang and Xu, 2008). The transcription of HSP genes is controlled by heat stress transcription factors (Hsfs), which play a prominent role in thermo tolerance (Kotak et al., 2007). The recent identification of 22 Hsfs genes in the chickpea genome (both desi and kabuli) has provided valuable information on thermo tolerance in chickpea (Chidambaranathan et al., 2018). Quantitative PCR (Q-PCR) expression analysis of Hsfs in heat-stressed (> 35°C for 3 h) chickpea at two stages of development (15-day-old seedlings and during podding) revealed that *CarHsfA2, A6*, and *B2* were up-regulated at both the stages of growth and four other Hsfs (*CarHsfA2, A6a, A6c, B2a*) showed early transcriptional up-regulation (Chidambaranathan et al., 2018). A previous study identified three distinct classes of Hsfs (A, B, and C) (Lin et al., 2014).

Various other heat-responsive proteins induced by heat stress (42/25°C for 8 days), exclusively in the heat-tolerant chickpea genotype, may play a vital role in heat tolerance (Parankusam et al., 2017). A recent study identified a set of 482 heat-responsive proteins and several metabolic proteins, including phenylalanine ammonia lyase 2-like, pectinesterase 3, cystathionine gamma-synthase, monodehydroascorbate reductase, adenosyl methionine synthase, NADH dehydrogenase subunit, cytochrome b6, inositol-3-phosphate synthase, RNA polymerase, and ATP synthase subunit alpha protein that were strongly related to the heat response in chickpea (Parankusam et al., 2017). Understanding the differential role and expression of these proteins in chickpea genotypes will provide an important vision for mechanisms that confer thermotolerance in chickpea.

Transcription factors (TFs) play an important role in modulating cellular responses under different stress conditions by activating the transcription of target genes. WRKY TFs are a major family of transcriptional regulators in plants that influence the stress tolerance mechanism and form an integral part of cell signaling pathways (Agarwal et al., 2011; Chen et al., 2012). In chickpea, TFs for heat tolerance have been reported [*CaMIPS1* and *CaMIPS2* (Kaur et al., 2008b) and Ca_02170, Ca_16631, Ca_23016, Ca_09743, Ca_25602] (Agarwal et al., 2016). Recently, a genome-wide analysis of a WRKY TF gene model revealed the presence of 78 WRKY TFs evenly distributed across eight chromosomes in chickpea (Kumar et al., 2016). Car-WRKY TF is reportedly multi-stress responsive, playing a central role in stress signal transduction pathways (Konda et al., 2018). In the chickpea genome, seven genes were identified based on homology, *PIE1* (photoperiod independent early flowering 1), *ARP6* (actin-related protein), two *SEF* (serrated leaf and early flowering), and three *H2AZs* (histone 2A variant-Z, a thermosensor in plants) and analyzed for expression under heat stress (37°C) that are homologous to chromatin remodeling complexes (SWR1) in *Arabidopsis* (Chidambaranathan et al., 2016). Of the seven genes, *PIE1* was up-regulated during podding but downregulated at the seedling stage. Higher tissue-specific expression of *PIE1* and *SEF* genes was observed in root, flower, pod wall, and grain tissues than in shoots. During pod development, all three *H2AZ* genes might function as thermosensors, with greater downregulation within 15 min, 1 and 6 h of the heat stress treatment (Chidambaranathan et al., 2016).

#### Mechanisms For Improving Heat Tolerance

The damage from high-temperature stress mainly depends on the plant's defense response and the growth stage at the time of exposure (Farooq et al., 2017). Chickpea plants use adaptive strategies to avoid, escape, and tolerate heat stress (Wery et al., 1993; Toker et al., 2007). Leaves avoid the heat by changing orientation, reducing transpiration, and reflecting light (Wery et al., 1993). In heat-stressed chickpea plants, phenology was accelerated as days to flowering and podding decreased significantly at 35/20°C (Kaushal et al., 2013), which also reduced total plant biomass. Therefore, accelerated phenology may be detrimental to chickpea production and considered an escape mechanism. Early maturation is closely correlated with reduced yield losses (Jumrani et al., 2017). In chickpea, a simple and cost-effective field screening method for heat tolerance at the reproductive stage was developed by delayed sowing (Krishnamurthy et al., 2011), which enable the plants to expose to high temperatures (>35°C) during reproductive phase; accordingly, the number of filled pods per plant in late-sown crop as identified as a selection criterion for reproductive-stage heat tolerance. Recent research has suggested that heat stress tolerance indices mean productivity, geometric mean productivity, yield index, tolerance index (TOL), superiority measure, and stress susceptibility index can be used to identify chickpea genotypes based on grain yield under normal and heat-stressed conditions. Based on these selection indices, RVG 203, RSG 888, GNG 469, IPC 06-11, and JAKI 9218 had moderate to high heat tolerance (Jha et al., 2018a). Using a heat tolerance index (HTI), ICC 3362, ICC 12155, and ICC 6874 were identified as heat-tolerant lines (Krishnamurthy et al., 2011). Upadhyaya et al. (2011) identified ICC 14346 as a heat-tolerant genotype among 35 early maturing germplasm under ideal crop management (irrigation, nitrogen application) conditions in a field screening at Patancheru (India), based on grain yield (kg ha^–1^). The pollen selection method and pollen viability were used to confirm the heat tolerance in ICCV 92944 (Devasirvatham et al., 2012), ICC 1205, and ICC 1561 (Devasirvatham et al., 2013). Heat-tolerant chickpea genotypes are listed in **Table 1**.

**Table 1:** List of chickpea genotypes tolerant to heat, cold, and drought stress.

Abiotic stress	Donor parents	Basis of tolerance	Subject involved	Reference
**Heat stress**	ILC 482, Annegiri, ICCV 10	Higher cell membrane stability	Plant physiology	Srinivasan et al. (1996)
	ICCV 88512, ICCV 88513	Reproductive biology	Plant physiology	Dua (2001)
	ACC 316 and ACC 317	Early phenology	Plant physiology	Canci and Toker (2009)
	ICC 1205	Reproductive biology	Plant physiology	Devasirvatham et al. (2010)
	ICC 4958, ICC 14778, ICC 1205, ICC 456	Increased plant yield	Plant breeding	Krishnamurthy et al. (2010)
	ICC 14346	Early phenology	Plant physiology	Upadhyaya et al. (2011)
	Pusa 240, JG 218, ICCV 92944	Low yield reduction under heat	Plant breeding	Kumar et al. (2012a)
	RAU 52, HK 94-34,IPC 98-12,	stress		
	CSG 8962, GCP 101, Pusa 209, GNG 663			
	ICC 1205 and ICC 15614	Higher pollen viability, and pollen tube germination	Plant breeding and physiology	Devasirvatham et al., 2012, Gaur et al. (2012) Devasirvatham et al. (2013)
	ICC 15614, ICCV 92944	Reproductive biology	Plant physiology	Kaushal et al. (2013)
	ICCV 07110, ICCV 92944	Biochemical	Plant biochemistry	Kumar et al. (2013)
	BG 256	Yield related traits	Plant breeding	Jumrani and Bhatia (2014)
	Katila, Vaibhav, Avrodhi	Yield related traits	Plant breeding	Jha and Shil (2015); Jha et al. (2015)
	GNG1958, ICC 15955, ICC1510	Heat tolerance indices based on	Plant breeding	Jha et al. (2017)
		yield per plant		
	IPC 2010-62, BRC 2, GNG 2215	Yield related traits	Plant breeding	Kumar et al. (2017)
	Pusa 1103, Pusa 1003, BGM 408, Pusa 240, PG 95333, JG14	Heat tolerance indices based on yield and physiological traits	Plant breeding and plant physiology	Kumar et al. (2017)
	PhuleG 13110, NBeG 507, BG3043	Pods/plant, yield/plant	Plant breeding	Agrawal et al. (2018)
	RVG 203, JAKI 9218, JG 130	Heat tolerance indices based on	Plant breeding	Jha et al. (2018a)
	ICCV0 7118, ICC1356	yield per plant		
	ICC 14778, ICC 15618	Yield related traits	Plant breeding	Varshney et al. (2019)
	ICC 96029	Early phenology (escape mechanism)	Plant physiology	Kumar and Rao (1996)
**Drought**	ICCV 2	Early phenology (escape mechanism)	Plant physiology	Kumar and Abbo (2001)
	ICC 5680, ICC 10448	Leaf trait	Plant physiology	Saxena (2003)
	ICC 4958	High root biomass, and volume	Plant physiology	Krishnamurthy et al. (2003) and
		deep rooting		Kashiwagi et al. (2005; 2006a)
	ICC 8261	Root trait (avoidance mechanism)	Plant physiology	Gaur et al. (2007)
	ICC 4958, ICC 8261	Root trait	Plant physiology	Kashiwagi et al. (2008)
	ACC 316 and ACC 317	Early phenology (escape mechanism)	-	Canci and Toker (2009)
	Gokce	High anti oxidant enzyme activity	Plant physiology	Macar and Ekmekci (2009)
		High proline and anthocyanin accumulation		
	MCC 544, MCC 696 and MCC 693	High proline accumulation	Plant biochemistry	Mafekheri et al. (2010)
	ICC 4958, HC 5	Maintains high photosynthesis rate	Plant physiology	Kumar et al., (2012c)
		and relative water content		
	ICC 7571	High harvest index	Plant physiology	Kashiwagi et al. (2013)
	Phule G 09103, Phule G 2008-74, Digiijay	Lower yield and chlorophyll, reduction and	Plant breeding and plant physiology	Ulemale et al. (2013)
		low membrane injury		
	FLIP03-145C, ILC 3182, and ILC 588	High yield and low days to maturity	Plant breeding and plant physiology	Hamwieh and Imtiaz (2015)
	FLIP03-100, FLIP05-123C,FLIP03-98	Based on drought tolerance indices	Plant breeding	Jha et al. (2016)
	IPC2009-102 and IPC2009-186			
	ICC 16374B, ICC 15510	Deep rooting that may help in accessing sub soil	Plant physiology	Chen et al. (2017)
	ICC9586 and ICC 867	moisture during drought stress		
	Neelam	High seed yield and	Plant physiology	Pang et al. (2017)
		conservative water use efficiency		
	DICC8172	Pod and seed	Plant physiology	Pang et al. (2017)
		Decrease in photosynthesis and		
		assimilate supply to seed		
	Bakhar-2011	Higher proline, total phenolics, and trehalose accumulation and stable carbon assimilation	Plant physiology and biochemistry	Farooq et al. (2018)
**Cold**	ILC 3470, FLIP 82-64C	Low yield loss	Plant breeding	Malhotra and Singh (1991)
	ILC 8262, ILC 8617,(FLIP 87-82C	Low yield loss	Plant breeding	Singh et al. (1995)
	*Cicer pinnatifidum, Cicer judaicum*			
	*Cicer echinospermum*			
	Sonali and Rupali	High viability and fertility of pollen	Plant physiology	Clarke et al. (2004)
	ICC 16348 and ICC 16349	Low electrolyte leakage,	Plant physiology	Kumar et al. (2011)
		low decrease in chlorophyll content		
	ICC16349	–	–	Sharma and Nayyar,(2014)
	Punjab 2008	Higher proline, total phenolics, and trehalose accumulation and stable carbon assimilation	Plant physiology and biochemistry	Farooq et al. (2017)
	PI 489777 (*Cicer reticulatum* Ladiz)	**–**	Plant breeding	Mugabe et al. (2019)

Various physiological traits—such as stomatal responses, membrane thermostability, chlorophyll fluorescence (CFL), canopy temperature depression (CTD)—have been associated with heat tolerance (Priya et al., 2018). Stomatal responses to heat stress is one possible mechanism for heat adaptation in chickpea; in a recent study, stomatal conductance and leaf water content (RWC) were significantly lower in heat-sensitive genotypes, relative to the unstressed plants, and significantly higher in tolerant genotypes, when grown under HS environment (>32/20°C) (Kaushal et al., 2013). Therefore, it can be assumed that stomatal conductance plays an important role during heat stress. Membrane thermostability is another important trait for heat tolerance, which has been considered a possible selection criterion for heat tolerance in chickpea, faba bean, and lentil based on electrolyte leakage from the leaves (Ibrahim, 2011). When tissues are subjected to high temperatures, electrical conductivity increases due to damage to cell membranes, consequently resulting in solute leakage. Electrolyte leakage increased under high temperature (>32/20°C) in a heat-sensitive chickpea genotype, relative to a heat-tolerant genotype (Kaushal et al., 2013; Parankusam et al., 2017). Thermal techniques have been used to measure canopy temperature; genetic variability in CTD (canopy temperature depression) was reported in chickpea under high temperature (32–35°C) (Devasirvatham et al., 2012), which correlated with yield. The genotypes with lower CTD (1–3°C) had lower grain yields than those with higher CTD (> 4°C) (Devasirvatham et al., 2015).

### Effects of Drought in Chickpea

Chickpea is predominantly grown in resource-poor, arid, and semi-arid regions under rainfed conditions. Consequently, drought stress can decrease chickpea yields by up to 50% (Sabaghpour et al., 2006). Drought stress impairs key physiological and biochemical processes ranging from photosynthesis, CO_2_ availability, cell growth, respiration, stomatal conductance, to other essential cellular metabolisms (Mansfield and Atkinson, 1990; Chaves, 1991; Chaves et al., 2003; Flexas et al., 2005; Chaves et al., 2009; Pinheiro and Chaves, 2011).

In subtropical (South Asia and north-eastern Australia) and Mediterranean climatic regions (such as southern Australia), chickpea faces “terminal drought” during the reproductive phase (Leport et al., 1999; Siddique et al., 1999), which can seriously impair reproductive processes, viz. anthesis, pollination, and also causes malfunction of reproductive organs especially pollen germination, pollen viability, fertility, and pollen tube growth and even dysfunction of stigma and style (Leport et al., 1998; Leport et al., 1999; Pang et al., 2017). However, drought stress at young plant stage or prior to reproduction is not uncommon. Drought at young plant stages reduces plant growth leading to stunting and reduced biomass accumulation (Siddique et al., 1999). Water deficit during podding in chickpea increased ABA that may impair pod set and cause pod abscission which can ultimately cause significant yield losses (Pang et al., 2017). Drought stress in chickpea can also lead to the collapse of symbiotic N2 fixation processes, resulting in serious yield losses (Wery et al., 1993).

#### Genetic Variability for Capturing Drought Stress Tolerance in Chickpea

The exploitation of natural genetic variation across various crop gene pools remains central to improving drought stress tolerance in crops, including chickpea. Considerable genetic variability for drought stress tolerance in chickpea has been recorded for various morpho-physiological and grain yield-related parameters under contrasting water regimes in the field (Krishnamurthy et al., 2010; Jha et al., 2014; Pang et al., 2017). Simple field-based screening techniques and superior crop yield performance has identified several chickpea genotypes under non-stressed and water stress conditions (Singh et al., 1997b; Toker and Cagirgan, 1998; Canci and Toker, 2009). Likewise, stress tolerance indices *viz.* drought susceptibility index and drought tolerance index, identified significant genetic variability for various phenological and yield-related traits under water stress in a large mini-core collection of 211 accessions (Krishnamurthy et al., 2010) (**Table 1**).

Considering the role of wild species as an important reservoir for imparting drought tolerance, *Cicer anatolicum*, *Cicer microphyllum*, *Cicer songaricum* are worth mentioning (Toker et al., 2007). Likewise, Kashiwagi et al. (2005) identified chickpea landraces in the Mediterranean, west Asian, and central Asian regions with high genetic variability for root length density that could be exploited for developing high water-use-efficient chickpea genotypes under water stress. Water use efficiency (WUE) is an important strategy for drought tolerance in crop plants, including chickpea (Condon et al., 2004; Zaman-Allah et al., 2011a; Zaman-Allah et al., 2011b), where a significant amount of genetic variability has been recorded (Pang et al., 2017). The authors identified “Neelam” as drought tolerant genotype, based on high WUE, as this genotype used a “conservative water use strategy” to maintain higher seed yields under water stress during early growth.

Root architecture traits are important parameters for improving crop performance under drought stress (Wasaya et al., 2018; Ye et al., 2018). Considerable progress has been made in elucidating the role of various root traits for drought stress tolerance in chickpea (Kashiwagi et al., 2006a; Kashiwagi et al., 2015). How root biomass, root length, and other root-related parameters, such as root length density (RLD), total root dry weight (RDW), and deep root dry weight (deep RDW), contribute to drought stress tolerance has been investigated in chickpea (Krishnamurthy et al., 2003; Kashiwagi et al., 2005; Gaur et al., 2008; Kashiwagi et al., 2008; Kashiwagi et al., 2015; Purushothaman et al., 2016; Chen et al., 2017). A significant amount of genetic variability for RLD in the mini-core collection and wild species of chickpea has been reported (Kashiwagi et al., 2005). Given their larger RLD, deep rooting system, and higher root biomass production, ICC 4958 and ICC 8261 genotypes are used extensively as donors for transferring important drought adaptive root traits to elite chickpea cultivars to develop drought-resilient chickpea cultivars (Saxena et al., 1993; Gaur et al., 2008). In addition, ICC 4958 remains one of the most extensively studied chickpea genotypes both in classical and modern molecular breeding programs for dissection of various traits, including drought-stress-related root traits.

Thus, these genotypes (ICC 4958 and ICC 8261) have been steadily incorporated into drought tolerance breeding programs for transferring the above-mentioned traits into elite chickpea varieties and developing mapping populations for deciphering drought-tolerant QTLs (Gaur et al., 2012). Concurrently, efforts are underway to develop multi-parent advanced generation inter-cross populations (MAGIC) by incorporating ICC 4958, JG 130, ICCV 10, JAKI 9218, JG 130, JG 16, ICCV 97105, and ICCV 00108, genotypes possessing drought and heat tolerance genomic regions/QTLs (Devasirvatham and Tan, 2018). Thus, selection from the resultant crosses could increase genetic gain in chickpea. Moreover, Chen et al. (2017) provided scope for improving drought tolerance in chickpea by investigating 30 root-related traits and three shoot-related traits in a large set of 270 core collection. ^13^C discrimination, an important physiological selection parameter related to water stress could also be used to enhance WUE under drought stress (Condon et al., 2002). A significant amount of genetic variability for ^13^C discrimination has been recorded in the chickpea reference germplasm collection (n = 280) (Upadhyaya et al., 2008; Krishnamurthy et al., 2013b).

Advancements in breeding techniques such as MAGIC have enabled the transfer of drought- and heat-tolerant traits into elite high-yielding chickpea cultivars by combining favorable allele combinations for drought and heat tolerance (Gaur et al., 2014; Gaur et al., 2019). Furthermore, marker-assisted recurrent selection (MARS) and marker-assisted backcrossing (MABC) efforts have been successfully used to transfer a “*QTL-hotspot*” genomic region harboring important drought-tolerant-related traits from donor parent ICC 4958 to JG 11 elite cultivar (Varshney et al., 2016).

#### Role of Physiological Traits for Adaptation Under Drought and Heat and Increasing Future Genetic Gain in Chickpea

Direct phenotypic selection for yield and yield-related traits has led to ignoring various important physiological traits that have great potential for increasing genetic gain and significantly contributing to plant acclimation under various abiotic stresses (Reynolds and Langridge, 2016). The incorporation of “physiological traits” in crop breeding programs provides an opportunity to enhance the chances of “cumulative gene action for yield” (Cossani and Reynolds, 2012). However, the success of incorporating various physiological traits depends on how the traits are associated with grain yield, their heritability, their ease of selection response and measurement, and their non-destructive nature (Monneveux et al., 2012).

Plant withstand drought and heat stress by recruiting “escape,” “tolerance,” and “avoidance” mechanism (Levitt, 1972). In the context, the major physiological traits involved in drought stress adaptation are categorized into “constitutive traits” and “acquired tolerance traits” (Sreeman et al., 2018). The notable “constitutive traits” involved in drought stress adaptation in chickpea include phenology (Kumar and Abbo, 2001), stomatal conductance (Liu et al., 2003), specific leaf area (Purushothaman et al., 2016), leaf area index (Purushothaman et al., 2016), chlorophyll content (Mafakheri et al., 2010), WUE (Kashiwagi et al., 2006b), and root traits (Krishnamurthy et al., 2003; Gaur et al., 2008; Kashiwagi et al., 2006a; Kashiwagi et al., 2015; Zaman-Allah et al., 2011b; Purushothaman et al., 2015). Likewise, canopy temperature depression (CTD) (Zaman-Allah et al., 2011a; Purushothaman et al., 2016), proline accumulation (Macar and Ekmekci, 2009; Mafakheri et al., 2010), regulation of ABA (Pang et al., 2017), and production of various antioxidant scavenging enzymes (Macar and Ekmekci, 2009) are the major “acquired tolerance” traits involved in drought stress tolerance in chickpea.

Prioritizing early phenology traits, *viz*. selection for early flowering and maturity, helps in the selection of genotypes exhibiting drought and heat stress tolerance in the form of an escape mechanism (Canci and Toker, 2009; Gaur et al., 2012; Hamwieh and Imtiaz, 2015). Relying on this mechanism important drought tolerant varieties viz., ICCV 90629, ICCV 2, ICCC 37, ICCV 10 (Kumar and Abbo, 2001), KAK2 (Gaur et al., 2008), and heat tolerant variety ICCV92944 (Gaur et al., 2012) were developed, however they suffered yield penalty due to restricted photosynthetic period, rapid growth rate, high harvest index, and short lifecycle (Kashiwagi et al., 2015; Berger et al., 2016).

#### Shoot Related Traits Contributing in Drought Stress Tolerance

Stomatal conductance (g_s_) is an important shoot-related parameter affecting leaf gas and water vapor exchange under stress conditions. Drought stress negatively affects stomatal conductance and leaf turgor (Liu et al., 2003). Zaman-Allah et al. (2011a) and Pang et al. (2017) argued genotype having lower stomatal conductance and utilizing lower water during vegetative stage at well-watered condition displayed higher drought tolerance at reproductive stage by using the conserved soil water at “terminal drought” stress. However, this “water sparing” will be effective for the crops those grow under stored soil water condition (Vadez et al., 2012). Insight into the genetic inheritance of stomatal conductivity and selection for lower stomatal conductance with higher leaf transpiration efficiency under drought could be promising for the development of drought tolerant chickpea genotypes. Likewise, correlations between crop growth rate and transpiration and transpiration efficiency are receiving attention in the development of drought-tolerant chickpea (Purushothaman et al., 2016).

Among the various non-destructive physiological traits, CTD infrared thermometer based parameter acting as a surrogate trait for transpiration explains the difference between air temperature [*T*a] and canopy temperature [*T*c] (Balota et al., 2007). It has received great attention as a potential selection tool and is regularly employed for screening high yielding drought and heat stress tolerant plants (Mason and Singh, 2014). This parameter depicts plant transpiration status that plays an important role in reducing leaf temperature under both drought and heat stress. Lower canopy temperature is indicative of higher transpiration, which enables plants to maintain their water status for growth under heat stress and water stress (Zaman-Allah et al., 2011a). In this context, a positive association of CTD with grain yield was noted under heat stress (Devasirvatham et al., 2015) and under drought stress (Purushothaman et al., 2015) in chickpea. Likewise, under drought stress, cooler canopy temperatures enhance root biomass, root depth, and ultimately grain yield (Lopes and Reynolds, 2010). Thus, further research of CTD at a genetic level could give better insight how to use this traits to develop drought and heat stress tolerance chickpea genotypes.

#### Role of Water Use Efficiency in Drought Stress Adaptation

WUE defines “biomass accumulated in plant at the cost of per unit water transpired” (Bacon, 2004). An array of traits ranging from stomatal regulation, transpiration rate to root traits could be employed for increasing WUE. Regulation of stomatal opening remains a great paramount importance, as restriction in stomatal opening increases reduction in transpiration leading to enhance WUE (Saradadevi et al., 2017). In this context, Zaman-Allah et al. (2011a) opined that lower stomatal conductance and lower transpiration could save water to be utilised during reproductive period under “terminal drought” stress in chickpea. However, reduction in stomatal opening causes lower intake of CO_2_ that may lead to decrease in photosynthetic carbon accumulation (Vadez et al., 2012). This mechanism of water stress tolerance works well when chickpea is grown in high water holding capacity soil in the south and central India featuring warmer and shorter growing period for chickpea (Berger et al., 2006; Berger et al., 2016). Contrastingly, high transpiration rate, high above and below ground biomass, high seed yield are the characteristics features of chickpea when it is grown under high rainfall receiving areas viz., northern Indian condition with low water holding capacity and with later phenology (Berger et al., 2006; Berger et al., 2016). Relying on the result explaining positive correlation of WUE with biomass yield under drought stress, Wright (1996) argued that increase in WUE could promisingly enhance plant yield provided harvest index is maintained.

Likewise, carbon isotope discrimination (Δ^13^C) is a noteworthy physiological attribute for measuring transpiration efficiency/WUE of plants under drought or heat stress. Kashiwagi et al. (2006b) suggested a negative correlation between Δ^13^C and WUE. However, its high cost of measurement remains a barrier to measuring WUE in larger numbers of genotypes. Thus, future genetic and molecular studies targeting traits improving WUE and optimizing transpiration rate could be beneficial in developing drought tolerant chickpea cultivars.

#### Role of Root Traits Contributing Drought Adaptation

Root system architecture is an important parameter that directly controls plant water content, which influences crop performance under water stress (Ye et al., 2018). Besides, root senses drought stress under dry soil and signals to produce ABA that causes closure of stomata resulting restriction of water loss through transpiration (Saradadevi et al., 2017). The crucial role of root traits, *viz.* RLD, root biomass, total RDW, root diameter, root volume, and root surface area, in controlling plant water status and how they help chickpea to adapt to water stress has been investigated (Krishnamurthy et al., 2003; Gaur et al., 2008; Kashiwagi et al., 2006a; Zaman-Allah et al., 2011b; Kashiwagi et al., 2015; Purushothaman et al., 2015). Mostly root traits play critical role in drought adaptation in chickpea by facilitating mining water through deep root and minimizing transpiration under water stress (Berger et al., 2016). In order to elucidate the role of root traits contributing in grain yield, Gaur et al. (2008) showed higher RLD and maximum root depth (RDp) in shallow soil could assist in increasing seed yield under drought stress. Likewise, Ramamoorthy et al. (2017) also evidenced positive association of RLD and grain yield under drought stress in chickpea. However, positive association of root traits with grain yield under drought stress remains inconsistent across various environment (Zaman-Allah et al., 2011b), leading plant breeders reluctant to use this trait in breeding program for drought tolerance. Thus, under central and south Indian condition where chickpea faces “terminal drought” stress, root traits based on “drought avoidance” strategy could be a promising approach for designing drought tolerant chickpea varieties (Kashiwagi et al., 2015). However, when chickpea grown under “in-season rainfall” in low water holding capacity soil under Mediterranean climates in Western Australia, this “drought avoidance” strategy remains ineffective (Berger et al., 2016).

#### Response of Biochemicals Alleviating Drought and Heat Stress

Plants including chickpea maintain turgor pressure and cell wall plasticity under water stress through recruiting osmotic adjustment mechanism that allows accumulating crucial biochemical compounds, including proline, glutathione, trehalose, molecular chaperones, and various antioxidant enzymes (Macar and Ekmekci, 2009; Mafakheri et al., 2010; Kaushal et al., 2011; Berger et al., 2016; Kaur et al., 2017; Farooq et al., 2018). Among the various stress-responsive chemical compounds, proline remains a critical amino acid produced in plants in response to stress. The differential expression pattern of proline synthesis enzyme (Δ1-pyrroline-carboxylate synthetase) and catabolism of proline by proline dehydrogenase in response to water stress at different vegetative and reproductive stages in drought-tolerant and drought-sensitive genotypes has been investigated in chickpea (Kaur et al., 2017). The desi Bakhar-2011 chickpea genotype accumulated more proline, trehalose, and non-reducing sugars to tolerate drought stress more than Bitall-2016 desi genotype by alleviating the adverse effects of oxidative stress and maintaining better carbon assimilation (Farooq et al., 2018). Likewise, to detoxify and to protect cellular damage from reactive oxygen species (ROS) viz., superoxide radicals, singlet oxygen accumulating under drought and heat stress, several ROS scavenging anti-oxidant enzymes such as superoxidase dismutase, catalase, glutathione peroxidase are worth mentioning biochemicals that enable chickpea adapting under drought and heat stress (Mafakheri et al., 2011; Kaur et al., 2017). Recently, Ullah et al. (2019) proposed that supply of zinc based nutrition could also assist in enhancing antioxidant activities and alleviate the detrimental effects of drought and heat stress in chickpea. These mechanisms are effective under moderate dehydrating conditions and impart partial drought tolerance (Farooq et al., 2018).

**Figure 1:**
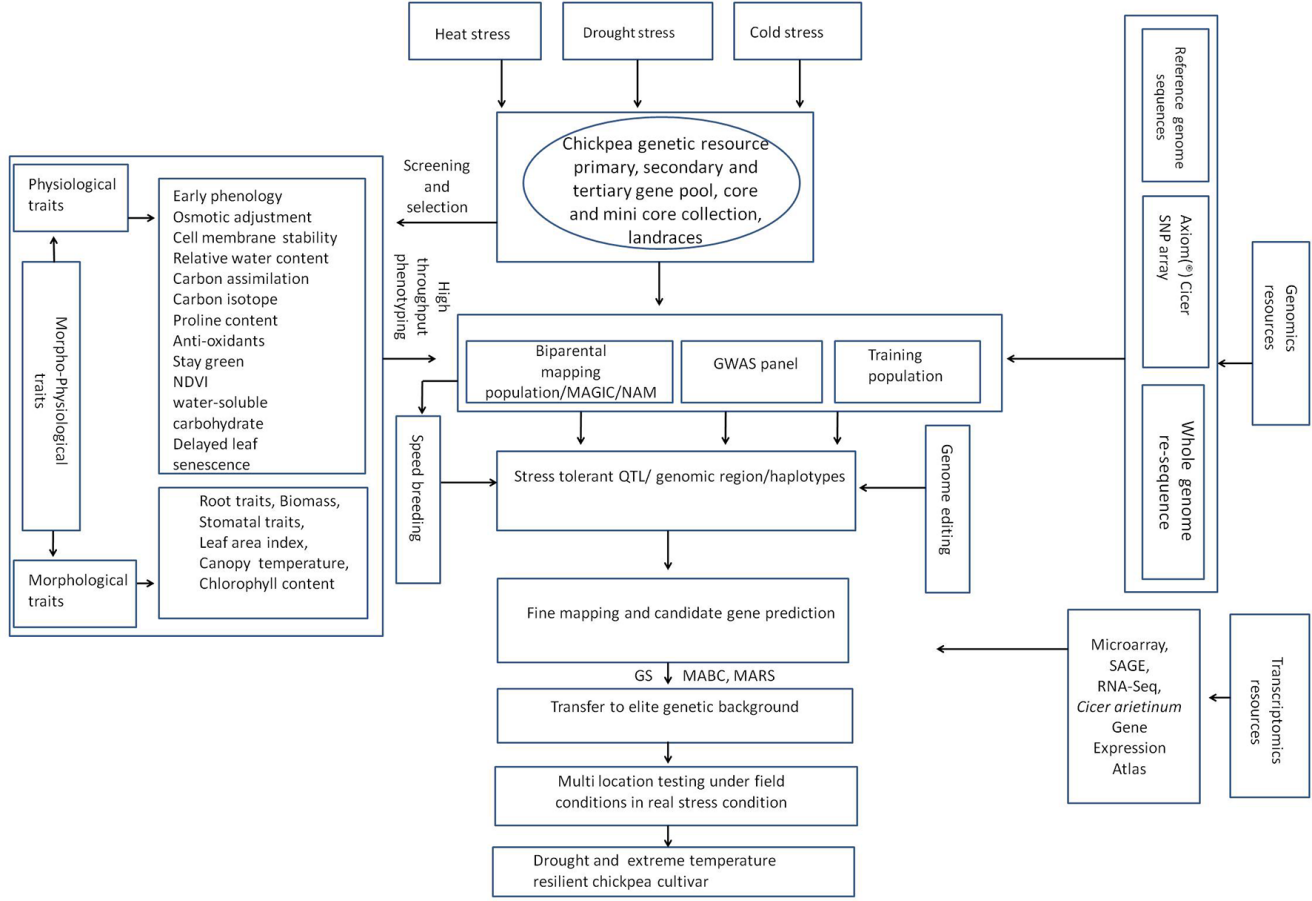
Integration of genomic approaches with physiological traits for breeding drought and temperature extreme resilient chickpea cultivar.

A holistic approach encompassing plant physiological approaches, genomics tools, and innovative breeding techniques for designing drought and extreme temperature tolerant chickpea cultivars has been depicted in **Figure 1**.

#### Advances in Genomics for Developing Drought and Heat Stress Tolerance in Chickpea

Investigating the genomic resources such as simple sequence repeat markers (SSRs) and single nucleotide polymorphism (SNPs) is vital for mapping of genes/QTL as well as for identifying genes related to drought and heat tolerance in QTL intervals. In the last decade, unprecedented advancements in molecular marker development and construction of high-density linkage maps have enabled precise mapping of various traits of breeding interest at specific locations across linkage groups in chickpea (Thudi et al., 2011; Jha et al., 2018b). Considering drought and heat stress tolerance, family-based bi-parental mating scheme derived mapping populations were limitedly devoted to elucidating QTLs controlling traits associated with various morpho-physiological and yield and yield-related traits under drought and heat stress in chickpea (Rehman et al., 2011; Hamwieh et al., 2013; Paul et al., 2018). However, the resultant QTL intervals remained large. Additionally, precise mapping of drought stress tolerance QTL remains challenging as it is controlled by various “minor effect QTLs” and remains unstable across the various locations due to high G×E interaction (Fleury et al., 2010). Increasing facilities of high density genotyping with large number of SSR markers and precise phenotyping of two mapping population segregating for various drought-related traits across multiple locations and multiple seasons allowed Varshney et al. (2014) to identify a “QTL-hotspot” harboring 13 main effect QTLs related to 12 drought-related traits, which explained up to 58% of the phenotypic variation on CaLG4. Subsequently, by adopting a marker-assisted backcross breeding scheme, this QTL-hotspot genomic region was introgressed from ICC4958 into JG11, an elite chickpea cultivar (Varshney et al., 2016). The resultant introgressed lines had greater root depth, RLD, and RDW (Varshney et al., 2016). However, this marker assisted breeding scheme remains effective for transferring “major effect QTLs” (Hayes et al., 2009). Further, advancements in next-generation sequencing technology (NGS) and high resolution genotyping platforms enabled the generation of huge numbers of SSR and SNP markers that assisted in narrowing the previously identified QTL-hotspot (Varshney et al., 2014) region to ~14 cM by recruiting genotyping-by-sequencing (Jaganathan et al., 2015). Furthermore, the combination of high density bin mapping and precise phenotyping of 17 drought-related traits across multiple locations and seasons further narrowed the QTL-hotspot region to ~300 Kb, and subdivided this genomic region into “*QTL-hotspot_a*” and “*QTL-hotspot_b*” regions on CaLG4 (Kale et al., 2015). Interestingly, QTLs contributing to plant vigor and canopy conductance under water stress were unfolded in this genomic region (Sivasakthi et al., 2018). Likewise, a total of four major QTLs developed from ICC 15614 × ICC 4567 RIL population controlling pod and grain yield trait were mapped on CaLG5 and CaLG6 under heat stress (Paul et al., 2018). Future cloning and functional characterization of these genomic regions could unravel the function of underlying gene(s), and thus facilitating designing of drought and heat stress tolerant chickpea genotypes.

Taking the advantage of higher resolution power of mapping complex QTLs owing to “natural evolutionary recombination events” genome-wide association study (GWAS) received great attention for unveiling “genotype-phenotype” associations elucidating the underlying novel candidate gene(s) controlling various complex traits including drought stress tolerance across large germplasm panel in various crop plants (Zhu et al., 2008; Huang and Han, 2014; Liu and Yan, 2019). In chickpea, GWAS has been used to better understand the genetic architecture of various complex traits of breeding importance [see Jha (2018)]. To elucidate marker-trait associations (MTA) for drought-related traits, Thudi et al. (2014) conducted GWAS in a large global collection of 300 chickpea genotypes. A total of 312 significant MTAs related to various drought and heat stress-related traits were identified providing a great opportunity for targeting those genomic regions for drought and heat stress tolerance breeding (Thudi et al., 2014). Similarly, five significant MTAs for cell membrane stability and chlorophyll content related to heat stress tolerance were deciphered from 71 chickpea genotypes containing historically released varieties of Indian and improved breeding lines (Jha et al., 2018b). Likewise, recently given the 3.65 million SNPs emanating from resequencing 429 globally collected chickpea germplasm, GWAS was used to elucidate significant MTAs for drought and heat stress tolerance in chickpea (Varshney et al., 2019). A total of 262 significant MTAs for various heat stress relevant traits, along with several potential candidate genes, *viz. TIC*, *REF6*, aspartic protease, *cc-NBS-LRR*, *RGA3* contributing in heat and drought tolerance were uncovered. Thus, the consistent and stable significant MTAs/genomic regions controlling pods/plant, yield trait, and phenological traits could be potentially incorporated in the high yielding yet drought/heat stress sensitive popular chickpea cultivars for improving drought and heat stress in chickpea.

Unparalleled advances in cost-effective genotyping platforms have enabled the generation of large-scale SNP marker information using WGS and WGRS of globally released chickpea cultivars, breeding lines, and germplasm accessions (Varshney et al., 2013b; Thudi et al., 2016; Roorkiwal et al., 2018a; Varshney et al., 2019). This has provided opportunities for the chickpea breeding community to use genomic selection (GS) (Meuwissen et al., 2001; Jannink et al., 2010) for various complex traits including drought stress tolerance (Roorkiwal et al., 2016; Li et al., 2018; Roorkiwal et al., 2018b). To date, several conventional breeding approaches have been devoted to increasing genetic gain by selecting superior individuals in chickpea under various biotic and abiotic stresses, including drought stress. However, this process remains slow due to yield and yield-related traits being governed by “small effect QTLs,” low heritability, and the influence of G × E interactions. GS could be one of the promising approaches to minimize this problem. GS constitutes “training population” with known genotypic and trait information, and is used to predict the genomic estimated breeding value of unobserved individuals of “candidate population” for complex traits with only genotypic information byusing various “trained statistical”/prediction models (Meuwissen et al., 2001; Jannink et al., 2010). Thus, the adoption of GS scheme could be a new avenue for capturing the “minor effect QTLs” across the whole genome and predicting increased genetic gain based on various prediction models under water stress in various crops, including chickpea (Hayes et al.2009; Crossa et al., 2017). The profuse numbers of SNP markers generated from 132 chickpea genotypes by WGRS allowed to conduct “SUPER GWAS” for unveiling the candidate genes associate to drought stress tolerance and also the sub set of SNPs were also used for performing GS for “prediction accuracy” of important yield related traits under drought stress (Li et al., 2018). Subsequently, Roorkiwal et al., 2018b investigated the implications of GS for precise prediction accuracy of genotypes incorporating G × E effects to enable selection of superior genotypes under various target environments for enhanced genetic gains in chickpea. However, the success of GS relies on high marker density, advanced genotyping platforms, heritability of trait, and optimization of the statistical model frameworks devised for GS (Roorkiwal et al., 2018a; Voss-Fels et al., 2019). Therefore, GS has great scope for selecting superior parents for crossing programs, maximizing selection accuracy, multi-trait selection in early generation, and speeding up the breeding cycle (Hayes et al., 2009; Jia and Jannink, 2012; Crossa et al., 2014; Crossa et al., 2017; Dias et al., 2018).

The arrival of NGS technologies in the last decade created a new dimension in genome sequencing chemistry, enabling the release of draft genome sequences of various plants of agricultural and economic importance (Michael and Jackson, 2013). The availability of draft genome sequences of kabuli (Varshney et al., 2013b), desi (Jain et al., 2013), and wild species (Parween et al., 2015) has sped up genomics research in chickpea. However, these genome sequences do not capture all the structural variations and presence–absence variation related to various traits. Falling cost of sequencing allowed us to sequence several genotypes/lines at a reasonable cost to capture the desired genomic regions. To obtain novel insight into drought-controlling genomic regions, WGRS of 100 chickpea genotypes has provided several important haplotypes that control drought stress tolerance (Thudi et al., 2016). Subsequently, Li et al. (2018) have unfolded significant associations of SNP markers released from WGRS of 132 chickpea lines with important drought tolerance candidate genes encoding auxin efflux carrier protein (PIN3), p-glycoprotein (PGP), and nodulin MtN21/EamA-like transporter. Recent efforts in WGRS of global chickpea germplasm coupled with GWAS have identified several drought-stress-controlling genomic regions (root traits, phenological traits, harvest index, 100 seed weight, delta carbon ratio etc.), including an important candidate gene *REF6* responsible for early phenology trait (Varshney et al., 2019). Further cloning and functional validation of this *REF6* gene and transfer of this gene through marker assisted breeding could help developing drought tolerant chickpea cultivar based on drought escape mechanism. Thus, translation of these genomics resources into applied breeding could expedite designing drought-tolerant chickpea varieties.

#### Functional Genomic Resources for Drought and Heat Stress Tolerance

Functional genomics remains a powerful approach for identifying the underlying candidate gene(s) and deciphering their functional role in response to various stresses including drought and heat stress in plant (Langridge et al., 2006). This approach can be employed in chickpea genotypes contrasting for stress sensitivity to obtain critical information about specific genes and their roles related to drought and heat tolerance. A significant progress in the development of genomic resources for dissection of drought and heat stress tolerance has been made (Varshney et al., 2014; Jaganathan et al., 2015; Kale et al., 2015; Varshney et al., 2016; Paul et al., 2018). However, the role of various candidate genes and their complex regulatory networks controlling drought and heat tolerance in chickpea at the functional level is limited (Hiremath et al., 2011; Agarwal et al., 2016; Garg et al., 2016); the information available about functional genomics largely pertains to drought tolerance.

Current advances in high throughput transcriptome sequencing technologies, especially RNA sequencing (RNA-seq), have provided novel insights into the molecular basis of drought tolerance by revealing the comprehensive landscape of divergent gene expression and their complex regulatory networks at various developmental stages at the transcriptional level (Garg et al., 2016; Kudapa et al., 2018). Before the advent of RNA-seq, microarray-based technologies and expressed sequenced tags (ESTs) were exclusively devoted to elucidating the preliminary function of various drought-stress-responsive genes/differentially expressed genes (DEGs) in chickpea (Mantri et al., 2007; Varshney et al., 2009; Deokar et al., 2011). Subsequently, given the RNA-seq driven global transcriptome analysis, a large number of water stress responsive DEGs (4954) were unearthed from root tissues of two contrasting drought tolerant (ICC 4958) and drought sensitive (ICC 1882) parents responding under water stress condition (Garg et al., 2016). Various DEGs identified under drought stress were found to be drought responsive TFs genes involved in controlling various hormone signaling ranging from abscisic acid, auxin, gibberellins, jasmonic acid, brassinosteroid to cytokinin (Garg et al., 2016; Badhan et al., 2018). Likewise, recently transcriptome sequencing of root and shoot tissue of two contrasting parents Bivanij and Hashem for drought resulted in 4,572 DEGs (Mahdavi Mashaki et al., 2018). From this investigation a total of seventeen common drought responsive genes from shoot and root were recovered. Importantly, to elucidate the role of candidate genes responding under drought stress, Bhattacharjee et al. (2015) reported higher up-regulatory role of *Ca_19899* (homeobox gene) in shoot tissue and down-regulatory role of *Ca_00550* gene both in root and shoot under drought stress. To mitigate the toxic effect of ROS activity produced under drought stress, Mahdavi Mashaki et al. (2018) unveiled up-regulatory activity of three genes (in Hashem) and *Ca_04125* gene (in Bivanij) involved in safeguarding cells against ROS toxicity. Likewise, up-regulatory activity of *Ca_05702* gene (participating in flavonoid biosynthesis), *CaNAC16 (Ca_18090*) (involved in water stress tolerance) and *Ca_00449* (carotenoid biosynthesis and producing ABA contributing in drought stress tolerance) in shoots of Bivanij under water stress were also substantiated (Mahdavi Mashaki et al., 2018). Additionally, participatory role of several TFs genes ranging from NAC, AP2/ERF, bHLH, WRKY, to MYB/MYC in essential metabolic pathways were also deciphered in chickpea under drought stress (Badhan et al., 2018; Mahdavi Mashaki et al., 2018; Kumar et al., 2019).

Furthermore, to identify the precise role of various candidate genes identified in the “hotspot QTL” region pinpointed by Kale et al. (2015) at the gene expression level, RNA-seq based global gene expression analysis revealed differential expression of nine candidate genes under water stress (Kudapa et al., 2018). Four genes namely *E3 ubiquitin‐protein ligase*, *LRX 2, kinase interacting (KIP1‐like) family,* and *homocysteine S‐methyltransferase*, displayed induced expression under drought stress (Kudapa et al., 2018). Likewise, RNA-seq analysis of various vegetative and reproductive tissues subjected to heat stress identified several important candidate genes, *viz. Ca_25811, Ca_23016, Ca_09743, Ca_17680*, contributing in heat-stress tolerance (Agarwal et al., 2016).

Similarly, non-coding RNA, including microRNA and long non-coding RNA (lncRNA), have received attention for their regulatory role in the expression of various genes controlling complex traits at the post-transcriptional level, including for drought stress in chickpea (Khandal et al., 2017; Singh et al., 2017). A microRNA (miRNA) profiling study of root apical meristem identified 284 unique miRNA sequences; of which 259 were differentially expressed under drought and salinity stress (Khandal et al., 2017). Functional validation of miRNA397 through qRT-PCR revealed its up-regulatory role under drought stress and it targeted *LACCASE4* gene that participate in lignin metabolism. To obtain deeper insight into the role of lncRNA for drought, a new tool “PLncPRO” was developed (Singh et al., 2017). A total of 3,714 lncRNAs involved in drought stress response in rice and chickpea have been discovered using this tool. However, the precise role of these lncRNAs in the drought stress response in chickpea and their functional annotation need further investigation. Further, availability of reference genome sequences, “*C. arietinum* gene expression atlas (CaGEA)” (Kudapa et al., 2018) and further refinement of transcriptome analysis could further increase our understanding of the complex drought and temperature stress responsive pathways, tracing the regulatory gene networks, and the underlying candidate gene(s), and their precise role in controlling drought and extreme temperature stress tolerances in chickpea. Moreover, transcriptome analysis could provide us great opportunity for revealing the genetic basis of higher adaptation of crop wild relatives (CWRs) and landraces to the counterpart of the cultivated species under various abiotic stresses (Srivastava et al., 2016). However, limited availability of abiotic stress tolerant cloned gene(s) has hampered the progress of functional genomics in chickpea (Deokar et al., 2015; Sen et al., 2017). Thus, in future mapbased cloning of abiotic stress tolerant gene(s)/QTLs could further illuminate our understanding of various mechanisms and key molecular players involved in drought, heat and cold tolerance in chickpea.

## Conclusion and Future Perspective

Current trends of unpredictable global climate change have resulted in periodic spells of drought stress and frequent episodes of extreme temperature, thus challenging plant growth and yield in several crops, including chickpea. Harnessing of crop germplasm, including various gene pools remains one of the most viable options in design of climate-resilient chickpea plants. *Cicer* cultigens are not adequately equipped with cold-tolerance; wild relatives *C. echinospermum* or *C. reticulatum*, the species of primary gene pool which are crossable to the cultigen, are however, good sources of cold tolerance. These species can be exploited to introgress cold tolerance to the cultigen. Incorporation of cold-tolerance in winter sown crop will lead to early flowering and maturity, a strategy that would allow the crop to avoid terminal drought, expected terminal high temperature due to global warming especially in winter/autumn sown crop and would increase reproductive period leading to enhanced productivity. Chickpea has indeterminate growth, and observations at two sites in north India (Palampur and Chandigarh, India) showed that temperature increase acts as a cue to terminate flowering and podding (Sharma and Nayyar, personal observations). If temperature remains conducive, chickpea plants would continue to flower and set pods due to indeterminate growth habit and this period can be increased by introgression of cold tolerance in chickpea. On the other hand, chickpea in warmer climates especially the spring-sown regions is expected to face higher terminal temperatures and high temperature tolerant chickpea must be developed for these regions for sustained productivity under global warming. Incorporation of drought tolerance in the cold tolerant as well as heat tolerant cultivars would be desirable as such dual tolerance chickpea would have additional protection from damage by drought apart from cold or heat stress.

Unlike cold-tolerance, heat-tolerant chickpea genotypes are relatively common to find in *C. arietinum*. In both types of temperature stresses, reproductive stage is the most sensitive one, and fails for similar reasons. Some cellular defense mechanisms such as osmolytes, carbohydrates, and antioxidants have been worked out by us under both heat and cold stress environments, which showed commonalities in their expression in responses to both the stresses but the picture fully clear in this context. Physiological mechanisms under combination of drought and heat as well as drought and cold are not fully understood. Further, it needs to be investigated whether heat-tolerant genotypes set pods under cold stress by subjecting them to LT under controlled environment, and testing their reproductive function and pod set. In case of cross tolerance, cellular defense mechanisms involving some stress-related metabolites and related genes may be probed to understand the underlying mechanisms. Since chickpeas have maximum acreage under rainfed and leftover soil moisture conditions and the crop invariably faces droughts at reproductive stage, this coupled with expected erratic rainfall under climate change scenarios warrants development of drought tolerant varieties. Terminal drought usually coincides with terminal heat stress in several chickpea growing regions, and hence, development of heat and drought tolerant chickpea cultivars is desired. Incorporation of various landraces and a range of crop gene pool harboring “adaptive traits” could enhance the resilience of chickpea genotypes under extreme climates.

Considerable understanding of physiological responses of genotypes of chickpea tolerant/sensitive to cold, heat, and drought is available, this understanding have, however, not been underpinned completely by the genetics/genomics. Genomics and transcriptomics have increased our understanding of gene and gene regulatory networks governing cold, drought, and heat stress, the understanding is, however, incomplete as it does not converge into well defined pathways governing tolerance or susceptibility to these three major abiotic stresses of chickpea. Unlike chickpea, we have considerably more information of plants' responses to various abiotic stresses in *Arabidopsis thaliana*. To identify well defined regulatory pathways for abiotic stress tolerance/sensitivity in chickpea, focus should be on establishment of role of individual genes identified through transcriptomics/genomics in tolerance or sensitivity and advancing this knowledge gradually to elucidate some specific as well as common responses of chickpea plants to these abiotic stresses. Owing to advancements in genomics in chickpea, QTLs/genes governing tolerance to the three abiotic stress traits and preliminary information on genes/gene interactions governing susceptibility/tolerance to these traits is available. The DNA-based markers, despite accelerated development during the last decade, are still inadequate and further enrichment of genomic resources for marker assisted selection is required so that adequately dense genetic maps be developed to map all the possible traits and narrow down the QTL boundaries in case of quantitative traits such as cold, drought, and heat stress tolerance. Considering drought stress, a “*QTL-hotspot*” harboring root and various drought related trait has been introgressed into elite chickpea genotype (Varshney et al., 2016). However, the other minor QTLs need to be pyramided individually or in combination for developing drought and heat tolerant elite chickpea varieties. Chickpea breeders still rely primarily on phenotypic selection for progeny plants while marker assisted selection (MAS) remained an underutilized technology even for monogenic traits like *Fusarium* wilt. Similarly, gene/QTL pyramiding has not been exploited in chickpea. Clearly, marker technology in chickpea is still in the laboratory stage waiting to be exploited commercially. Nonetheless, genomic resources such as markers linked to phenotypic traits and genes governing several traits are already known and this knowledge is expanding rapidly e.g., sequencing and resequencing approaches have increased repertoire of SNP markers during the last decade. This information indicates toward possible exploitation of genomic selection for phenotypic traits for chickpea in future.

Future research must aim at developing designer chickpea cultivars that can tolerate combination of stress environments, such as heat and drought, and cold and drought, to expand its stress tolerance ability along with superior agronomic performance. Exploitation of genomics/transcriptomics/resequencing coupled with reference genome sequences in chickpea, are expected to enhance our understanding of cold, heat and drought stress tolerance that in near future will boost development of single- or multiple stress tolerant high-yielding chickpea cultivars suited to specific climatic niches. This knowledge may consequently result in development of better and economical stress management options based on chemical/agronomic means, apart from host resistance, to enable us to deal with unexpected climatic contingencies.

## Author Contributions

AR and KDS compiled information about cold stress, and PD and UJ about heat and drought stress. KHM and HN thoroughly edited the manuscript and gave their inputs in organizing the text.

## Conflict of Interest

The authors declare that the research was conducted in the absence of any commercial or financial relationships that could be construed as a potential conflict of interest.
